# LncRNA GSCAR promotes glioma stem cell maintenance via stabilizing SOX2 expression

**DOI:** 10.7150/ijbs.80873

**Published:** 2023-03-05

**Authors:** Xiulin Jiang, Yong Zhang, Yixiao Yuan, Zhixian Jin, Haoqing Zhai, Baiyang Liu, Yao Li, Chun Zhang, Min Chen, Yulin Shi, Dongming Yan, Jun Pu, Yongbin Chen, Cuiping Yang

**Affiliations:** 1 The International Peace Maternity and Child Health Hospital, School of Medicine, Shanghai Jiao Tong University, Shanghai 200030, China.; 2 Shanghai Key Laboratory of Embryo Original Diseases, Shanghai 200030, China.; 3 Key Laboratory of Animal Models and Human Disease Mechanisms of Chinese Academy of Sciences & Yunnan Province, Kunming Institute of Zoology, Kunming, Yunnan 650223, China.; 4 Department of Pathology, Cancer Hospital of China Medical University, Shenyang, Liaoning 110042, China.; 5 Kunming Medical University, Kunming 650101, China.; 6 Department of Neurosurgery, the First Affiliated Hospital of Zhengzhou University, Zhengzhou 450052, China.

**Keywords:** GSCAR, miR-6760-5p, SRSF1, SOX2, glioma stem cells (GSCs)

## Abstract

Gliomas are the most aggressive type of malignant brain tumors. Recent studies have demonstrated that the existence of glioma stem cells (GSCs) is critical for glioma recurrence, metastasis, and chemo- or radio-therapy resistance. Temozolomide (TMZ) has been used as an initial therapy for gliomas. However, the overall survival time is still limiting due to the lack of effective targets and treatment options. Therefore, identifying novel biomarkers for gliomas, especially for GSCs, is important to improve the clinical outcome in the future. In this study, we identify a human-specific long non-coding RNA (lncRNA, ENSG00000250377), termed GSCAR (glioma stem cell associated lncRNA), which is highly expressed in glioma cancerous tissues and cell lines. We reveal that GSCAR positively correlates with tumor grade. Glioma patients with GSCAR high expression exhibit shortened overall survival time, compared to patients with GSCAR low expression. Furthermore, we show that GSCAR knockdown by shRNAs or antisense oligonucleotide (ASO) reduces tumor cell proliferation, migration and xenograft tumor formation abilities. Mechanistic study shows that GSCAR acts as a ceRNA (competing endogenous RNA) for miR-6760-5p to promote the expression of oncogene SRSF1 (serine and arginine rich splicing factor 1). In addition, GSCAR mediates the protein complex formation between DHX9 (DExH-Box helicase 9) and IGF2BP2 (insulin-like growth factor 2 mRNA-binding protein 2), leading to the stabilization of SOX2 (sex-determining region Y-box 2) mRNA and then the transcriptional activation of GSCAR. Depleting GSCAR reduces SOX2 expression and GSC self-renewal ability, but promotes tumor cell responses to TMZ. These findings uncover that GSCAR/miR-6760-5p/SRSF1 axis and GSCAR/DHX9-IGF2BP2/SOX2 positive feedback loop are critical for glioma progression, which could be used as prognostic biomarkers and therapeutic targets in the future.

## Introduction

Gliomas account for approximately 30% of all brain tumors, and pilocytic astrocytoma (WHO grade I) is the least malignant subtype, which can progress to most malignant glioblastoma (GBM, WHO grade IV), and the average survival time for GBM patients is approximately 15 months after diagnosis 1. Gliomas are characterized by intense neovascularization with unusual vessel-like structures and are commonly resistant to radio- or/and chemotherapies, which leads to tumor relapses and poor prognosis. During the past decades, dedicated studies in gliomas have resulted in the identification of multiple key genetic and molecular underpinnings, which contribute to the new classification for gliomas 2. Mutations in the IDH1/2 have been identified in gliomas, and IDH-mutant low-grade gliomas (LGGs) may develop malignant transformation after further genetic alterations, such as Myc, PTEN, KRAS, PIK3CA, and MET, are acquired. However, the pathological consequences resulting from IDH mutation remain elusive 3. To date, surgical resection, temozolomide (TMZ)-dependent chemotherapy, radiotherapy, and bevacizumab treatment are the conventional therapies for gliomas, which are still far from sufficient in combating tumor progression 4.

The different locations in the brain and the regulatory molecular events may generate various types of neural stem and progenitor cell (NSPC) pools, and glioma stem cells (GSCs) with self-renewing and tumorigenic abilities have also been identified, which are resistant to standard chemo- and radio-therapies, indicating their critical role in tumor recurrence and metastasis 5. Our group and others have recently identified that GSCs develop multiple molecular mechanisms to mediate therapeutic resistance, including hypoxia, Notch, EZH2, and DNA damage checkpoint-related signaling pathways 6, 7. Multiple biomarkers for GSCs, including SOX2, CD133 and CD44, have been documented in recent years, although the underlying mechanisms by which these biomarkers are specifically induced in GSCs need to be unraveled 7, 8.

Recently, an increasing number of findings have shown that noncoding RNAs may serve as valuable therapeutic targets for glioma patients 9. LncRNA-HOTAIR was highly expressed in high-grade gliomas (HGGs), which correlates with a poor survival rate 10. The tumor suppressive lncRNA GAS5 was described to inhibit GSC maintenance via a miR-196a-5p/FOXO1 feedback loop 11, while FOXM1-AS was found to facilitate the interaction of ALKBH5 with FOXM1 nascent transcript, leading to GSC activation and glioma progression 12. The Sox2 gene has been well documented as a pluripotent factor essential for stem cell self-renewal and differentiation 13. Furthermore, increased SOX2 related to adverse clinical outcomes in glioma patients, suggesting that depleting of SOX2 may be a novel therapeutic approach to combat glioma 14. However, the posttranscriptional regulation of SOX2 by long noncoding RNAs in gliomas remains unclear.

Here, we identified a 676-bp lncRNA, termed glioma stem cell association long noncoding RNA (GSCAR; ENSG00000250377), that is upregulated in glioma cancerous tissues and cell lines, especially in GSCs, and is correlated with worse clinical outcomes. We demonstrated that GSCAR promotes the growth, migration, and invasion of glioma tumor cells by competing for endogenous miR-6760-5p to induce the expression of the oncogene SRSF1. In addition, we showed that GSCAR activates the self-renewal ability of GSCs by mediating DHX9 and IGF2BP2 complex formation, leading to the stabilization of the SOX2 transcript and tumor growth. Therefore, we decided to decipher the potential mechanism by which the GSCAR/miR-6760-5p/SRSF1 axis and GSCAR/DHX9-IGF2BP2/SOX2 feedback loop promote glioma progression, which may provide new therapeutic targets for glioma in the future.

## Methods and Materials

### Constructs

Independent GSCAR, SRSF1, DHX9, SOX2 and IGF2BP2-targeting shRNAs were connected to the pLKO.1 vector 15, and all the oligonucleotides are indicated in **Table S1**. Human GSCAR and SRSF1 cDNA was amplified by PCR and subcloned into the pCDH-MCSV-E2F-eGFP vector. Lentiviral vectors expressing Ctrl shRNA, GSCAR shRNA#1, GSCAR shRNA#2, SRSF1 shRNA#1, and SRSF1 shRNA#2 were cotransfected into HEK-293T cells with pMD2.G and psPAX2 plasmids (Addgene), lentiviruses were packaged. ASOs targeting GSCAR, control ASO, miR-6760-5p mimics, and inhibitors were ordered from Ruibo and transfected into cells using Lipofectamine 3000.

### Chromatin immunoprecipitation (ChIP) assay

ChIP assay was performed as previously documented 16. Briefly, 9x 10^6^ cells were harvested, and 5 μg of preimmune mouse IgG and anti-SOX2 antibodies were used for the ChIP reaction 16. The oligo sequences are provided in **Table S1**.

### Tissue microarrays

Glioma tissue microarrays comprised of 10 normal brain tissues and 60 glioma tissues annotated with clinical and pathological information (Wuhan Servicebio, IWLT-C-70GL61, China) were used to verify GSCAR expression via RNA *in situ* hybridization (ISH). All specimens were graded by the pathological and clinical stages **(Table S2)**.

### RNA pull-down assay

For *in vitro* RNA synthesis, the GSCAR fragment was connected to pcDNA3.1, the construct was then linearized, and the RNAs were transcribed with T7 RNA polymerase. The Pierce™ RNA 3' End Desthiobiotinylation Kit was used to biotinylate sense and antisense GSCAR RNAs. These RNAs were then incubated with GSCs cell extracts at 4 °C. Then using Elution Buffer to elute potential proteins. The obtained proteins were then examined by SDS-PAGE followed by immunoblot and mass spectrometry detection.

### RNA immunoprecipitation assay

9 x 10^6^ GSC cells were lysed in 1 ml of RIP lysis buffer supplemented with RNase inhibitors. The GSCs cell lysates incubated with beads coated with IgG, anti-IGF2BP2, or anti-DHX9 antibodies on a rotator at 4 °C overnight. The RNA-protein complexes were washed and then incubated with the Proteinase K digestion system. Protein-bound RNAs were finally extracted by RNA extraction methods and performed RT-PCR examination.

### RNA decay assay

3X10^4^ GSC cells were seeded in 6-well plates and treated with actinomycin D (5 μg/mL) at 0, 4, and 8 h time points, respectively. Total RNAs were then isolated by TRIzol and subjected to RT-PCR.

### Mass spectrometry analysis

Proteins bound on the streptavidin magnetic beads were eluted with the Elution Buffer of the Pierce™ Magnetic RNA-Protein Pull-Down Kit (17-700, Millipore). The recovered proteins were then examined by mass spectrometry detection. All experiments were performed on a Q-Exactive mass spectrometer with an ancillary EASY-nLC 1000 HPLC system (Thermo Fisher Scientific). The mass spectrometry instrument parameters were: MS1 full scan resolution, 70000 at m/z 200; automatic gain control target, 3 × 10^6^; maximum injection time, 120 ms. The candidate GSCAR interacting proteins were indicated in **Table S3**.

### Tumorsphere formation assay

Briefly, 3x10^4^ GSCs cells were transferred to 6-well plates, and the spheres were cultured for approximately 14 days, white-field images were collected, and the sphere numbers were quantified.

### Bioinformatics assay

Most statistical assays were examined using GraphPad Software 7 (GraphPad Software Inc., CA, USA). The expression of lncRNAs, microRNAs, and mRNAs in Gene Expression Omnibus (GEO), The Cancer Genome Atlas (TCGA), and the Genotype-Tissue Expression (GTEx) 17, 18, the survival curves for the prognostic analysis were generated via the Kaplan-Meier method 19. The KEGG pathway enrichment analysis was performed using the GSEA software 20. The specificity and sensitivity of GSCAR, SRSF1 and miR-6760-5p were assessed via receiver operating characteristic (ROC) curves, and the area under the curve (AUC) was quantified using the pROC R package. The correlation was analyzed by Pearson's correlation analysis. The significance of the data between two experimental groups was determined by Student's *t*-test, and multiple-group comparisons were analyzed by one-way ANOVA. *P* < 0.05 (*), *P* < 0.01 (**), and *P* < 0.001 (***), were significant.

## Results

### GSCAR was highly expressed in gliomas

**Figure 1:**
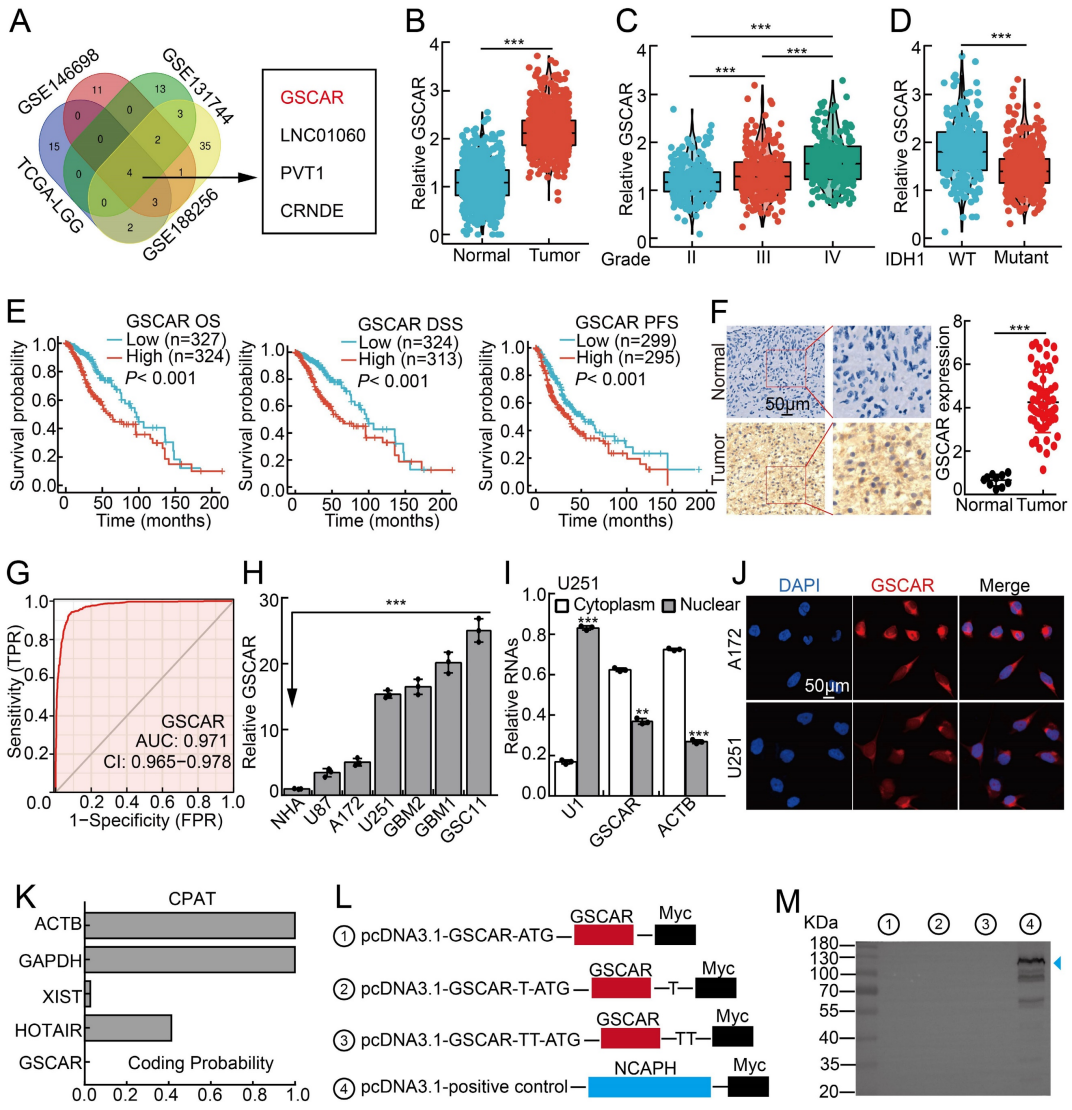
** GSCAR was highly expressed in gliomas. (A)** LncRNA GSCAR was identified by integrative omics analysis using GEO datasets, TCGA-LGG (blue): data generated from low-grade glioma tissue samples, GSE146698 (red): data generated from a TMZ-resistant cell line, GSE131744 (green): data generated from a glioma stem cell line, and GSE188256 (yellow): data generated from glioma tissue samples. **(B)** The relative expression levels of GSCAR in TCGA-LGG/GTEx datasets (Normal: 1152, Tumor: 523).** (C)** The relative expression levels of GSCAR in grade II, III, and IV glioma patients in the TCGA database (II: 224, III: 243, and IV: 168).** (D)** GSCAR expression was higher in IDH1 wild-type (WT: 246) patients than in IDH1 mutant (MUT: 440) patients. **(E)** High GSCAR expression correlates with a worse survival rate. OS: overall survival, DSS: disease-specific survival, and PFS: progression-free survival. **(F)** The expression of GSCAR in normal brain tissues and glioma tissues was examined by ISH assay (Normal: 10, Tumor: 60). Quantification results are shown. **(G)** The ROC curve for GSCAR (AUC=0.971) was examined by the TCGA glioma dataset. **(H)** The relative expression level of GSCAR in glioma cancerous cell lines (U87, U251, A172) and glioma stem cell (GSC) lines (GBM1, GBM2, GSC11). Fetal normal human astrocytes (NHAs) were used as controls. **(I)** GSCAR was primarily localized in the cytoplasm of U251 and A172 cells using the nuclear and cytoplasmic RNA fractionation assay followed by RT-PCR examination. ACTB (β-actin: cytoplasmic control), U1 (nuclear control). **(J)** The subcellular localization of GSCAR was examined by FISH assay. Scale bar=50 μm. **(K)** The coding probability of GSCAR was predicted by CPAT. **(L-M)** Full-length GSCAR was cloned into an eukaryotic expression vector pcDNA3.1 vector/myc with an N-terminal codon ATG in the three expression patterns. The blue arrowhead pointing to NCAPH-Myc proteins was used as a control. * *P* < 0.05, ** P < 0.01, *** *P* < 0.001.

To identify the potential oncogenic lncRNAs in gliomas, we characterized the lncRNAs located in SCNAs in gliomas using the TCGA-LGG dataset, and 24 candidate lncRNAs were selected according to the criteria (Relative CNAs in >40% glioma samples; occurring in the amplification CNA area; prior to long intergenic non-coding RNA; Log FC>4, *P*<0.0001). To narrow down the potent candidate involved in glioma stem cells, we further examined the deregulated lncRNAs in U251/TMZ (TMZ resistant cell line) and cancer stem cells **(Table S4)**. We uncovered that 4 lncRNAs were unanimously upregulated, including ENSG00000250377 (named GSCAR based on its functional role), LINC01060, PVT1, and CRNDE. Importantly, GSCAR, but not the other 3 lncRNAs, was identified as the only candidate whose functional role in gliomas remains elusive **(Figure 1A and Table S4)** 21-26.

We first found that GSCAR is specifically expressed in humans **(Figure S1A)** 27. We then confirmed that GSCAR expression positively correlated with SCNAs, which resulted in poor clinical outcomes **(Figure S1B-S1C)**. The increased expression of GSCAR in gliomas was validated in web-available datasets 28, and a significant correlation between high GSCAR expression and higher-grade tumors was detected **(Figure 1B-1C)**. Consistently, we found that GSCAR expression was higher in IDH1 wild-type (WT) gliomas than in IDH1 mutant (MUT) gliomas, and glioma patients with higher GSCAR expression exhibited worse clinical outcomes **(Figure 1D-1E)**. As expected, the increased expression of GSCAR was confirmed in glioma tissue microarray examined by ISH assay (RNA *in situ* hybridization) **(Figure 1F and Figure S1D)**. The ROC curve was applied to examine the diagnostic value of GSCAR in gliomas, which showed that the AUC value was 0.971, indicating that GSCAR may serve as an independent prognostic biomarker in gliomas **(Figure 1G)**. To corroborate the bioinformatics results, we then examined GSCAR expression in glioma cancerous cell lines and glioma stem cell lines GSC11, GBM1, and GBM2, and fetal normal human astrocytes (NHAs) were used as controls 7. We revealed that GSCAR was markedly upregulated in glioma cancerous cell lines and preferentially higher in GSCs **(Figure 1H)**. Consistent with the web-source dataset, we revealed that GSCAR was mainly located in the cytoplasm, which was further confirmed by the RNA FISH assay and the RT-PCR analysis after nuclear and cytosolic fractionation according to the documented references 29, 30 **(Figure 1I-1J and Figure S1E-1F)**. In addition, we uncovered that GSCAR could not be translated into coding-proteins using immunoblot following standard protocol **(Figure 1K-1 M)** 30, 31.

### GSCAR promotes tumor cell proliferation and migration

**Figure 2:**
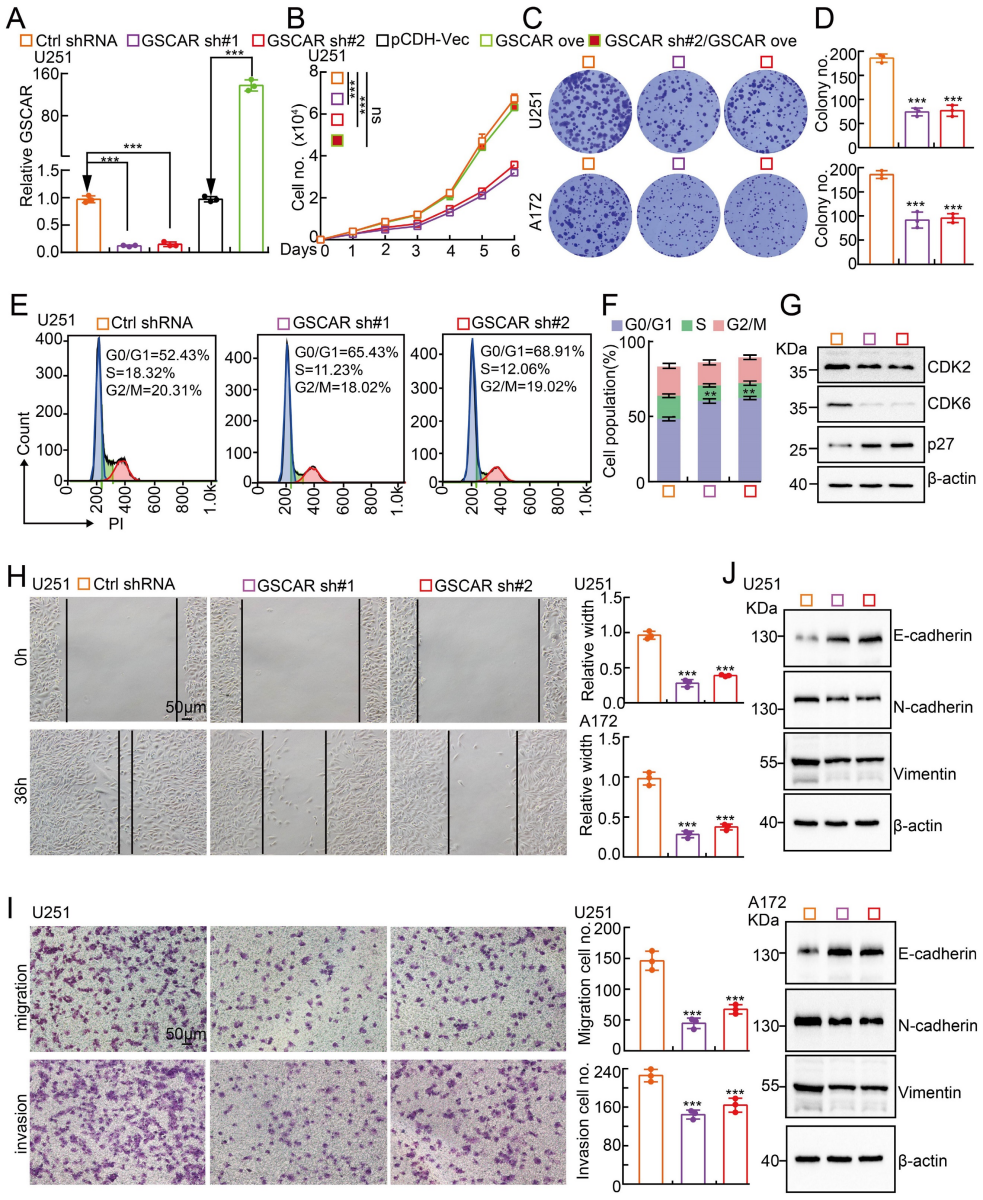
** GSCAR knockdown inhibited glioma cell proliferation and migration. (A)** Establishment of GSCAR knockdown and overexpression cell lines in U251 verified by RT-PCR assay.** (B)** GSCAR knockdown inhibited U251 cell growth. **(C-D)** Colony formation assay in U251 and A172 cells. (D) Quantification data for (C). **(E-F)** GSCAR knockdown blocked the G0/G1 cell cycle transition in U251 cells, as examined by PI staining and flow cytometry. (F) Quantification data for (E). **(G)** GSCAR knockdown reduced the protein expression of CDK2 and CDK6 while promoting p27 expression, as examined by immunoblot. The indicated cell extracts were probed with the indicated antibodies. **(H-I)** Knockdown of GSCAR inhibited U251 cell migration and invasion using wound healing (H) and transwell (I) assays. Quantification results are also indicated. Scale bar=50 μm. **(J)** The indicated cell extracts were probed with the indicated antibodies to examine the expression patterns of cell migration regulators, including E-cadherin, N-cadherin, and Vimentin. * *P* < 0.05, ** P < 0.01, *** *P* < 0.001. pCDH-Vec=pCDH lentiviral plasmid vector control. ove=overexpression, sh#1=shRNA#1, sh#2=shRNA#2. no.=number.

To investigate the biological function of GSCAR in gliomas, we first performed KEGG analysis to predict the potential molecular events regulated by GSCAR in low-grade gliomas (LGGs), and the cell cycle, Wnt signaling, and focal adhesion-related signaling pathways were identified **(Figure S2A)**. To validate the bioinformatics results, GSCAR was inhibited by specific shRNAs in U251 and A172 cells, and cells expressing scrambled shRNA were used as controls **(Figure 2A and Figure S2B)**. As expected, GSCAR knockdown inhibited tumor cell growth **(Figure 2B-2D and Figure S2C)**. The cell cycle transition was then detected by flow cytometry analysis, and we found that GSCAR knockdown caused an accumulated G0/G1 phase cell population compared to that in control cells **(Figure 2E-2F and Figure S2D-2E)**. Consistently, the critical factors regulating G0/G1 cell cycle transition, including CDK2 and CDK6, were markedly decreased, while p27 was increased, in GSCAR knockdown cells examined by immunoblot **(Figure 2G and Figure S2F)**. Furthermore, we found that GSCAR knockdown dramatically inhibited glioma cell migration and invasion abilities **(Figure 2H-2I and Figure S2G-2H)**. The expression of EMT-related genes was also verified by immunoblotting. As expected, E-cadherin was increased, while N-cadherin and vimentin were reduced upon GSCAR knockdown **(Figure 2J)**. These findings indicated that GSCAR functions as a potential oncogenic factor in gliomas.

### GSCAR competes with miR-6760-5p to induce oncogene SRSF1 expression

**Figure 3:**
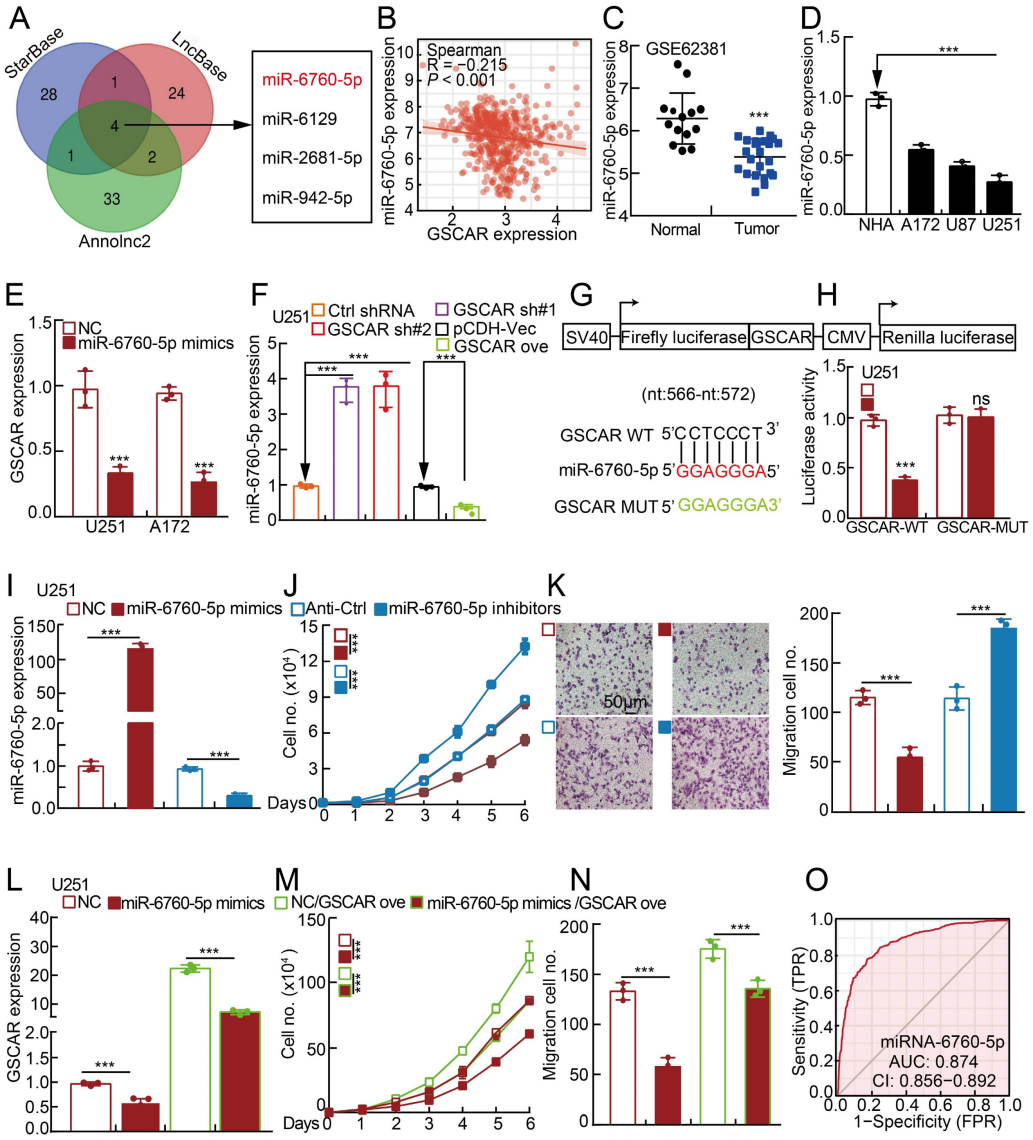
The **GSCAR/miR-6760-5p axis promoted glioma cell proliferation and migration. (A)** A total of 4 miRNAs were predicted to harbor complementary sequences to GSCAR using the StarBase, LncBase V2, and Annolnc2 datasets. **(B)** Correlation analysis between GSCAR and miR-6760-5p using the TCGA-LGG dataset. **(C)** The decreased expression of miR-6760-5p in the GEO dataset. **(D)** Relative miR-6760-5p expression in glioma cell lines compared to that in NHA cells detected by RT-PCR. **(E)** Relative expression of GSCAR with miR-6760-5p overexpression in U251 and A172 cells examined by RT-PCR. (**F**) Relative miR-6760-5p expression was examined by RT-PCR in the indicated cells.** (G)** A schematic picture of the wild-type (WT) and mutant (MUT) GSCAR luciferase reporter plasmids.** (H)** The luciferase activities of the GSCAR luciferase reporters (WT or MUT) were examined in U251 cells coexpressing miR-6760-5p mimics or NC. **(I)** Relative miR-6760-5p expression was examined by RT-PCR after transfection with the indicated oligos. **(J-K)** MiRNA-6760-5p mimic overexpression reduced, while miRNA-6760-5p inhibitor overexpression promoted, U251 cell growth (J) and migration (K). Quantification results are indicated.** (L)** Relative GSCAR expression was examined by RT-PCR in the indicated cells.** (M-N)** GSCAR overexpression overcame the cell growth and migration abilities repressed upon miR-6760-5p mimic overexpression. **(O)** The ROC curve for miR-6760-5p (AUC=0.874) in gliomas using the TCGA dataset. NC=negative control=miRNA mimic control, Anti-Ctrl=miRNA inhibitor control. * *P* < 0.05, ** P < 0.01, *** *P* < 0.001.

Based on the fact that GSCAR was mainly located in the cytoplasm, it is very likely that GSCAR mainly functions as ceRNAs. Therefore, we used StarBase, LncBase V2, and Annolnc2 to uncover the microRNAs (miRNAs) directly regulated by GSCAR 30, 32-35, and identified miR-6760-5p, miR-6129, miR-2681-5p and miR-942-5p as candidates **(Figure 3A and Table S5)**. To validate the functional microRNAs, we examined the candidate microRNA expression in gliomas. Only miR-6760-5p, but not all the other 3 microRNAs, was significantly negatively associated with GSCAR expression and was also markedly decreased in gliomas and cell lines **(Figure 3B-3D and Figure S3A-3B)** 32. Consistently, we showed that miR-6760-5p mimic overexpression in U251 and A172 cells markedly reduced GSCAR expression **(Figure 3E)**. Inhibition or forced expression of GSCAR increased or decreased miR-6760-5p expression, respectively, in glioma tumor cells **(Figure 3F and Figure S3C)**, which was validated by dual-luciferase reporter assay **(Figure 3G-3H and Figure S3D)**. To verify the functional role of miR-6760-5p in gliomas, miR-6760-5p mimics or inhibitors with reciprocal controls were then transfected into U251 and A172 cells. We detected that tumor cell growth and migration abilities were abrogated by miR-6760-5p mimics but were promoted by miR-6760-5p inhibitor overexpression **(Figure 3I-3K and Figure S3E-3G)**. In addition, the repressed cell proliferation and migration abilities resulting from miR-6760-5p mimic overexpression can be overcome by GSCAR overexpression, supporting the specific role of the GSCAR/miR-6760-5p axis in gliomas **(Figure 3L-3N and Figure S3H-3J)**. The ROC curve analysis of miR-6760-5p exhibited an AUC value of 0.874, supporting its prognostic value in gliomas **(Figure 3O)**.

**Figure 4:**
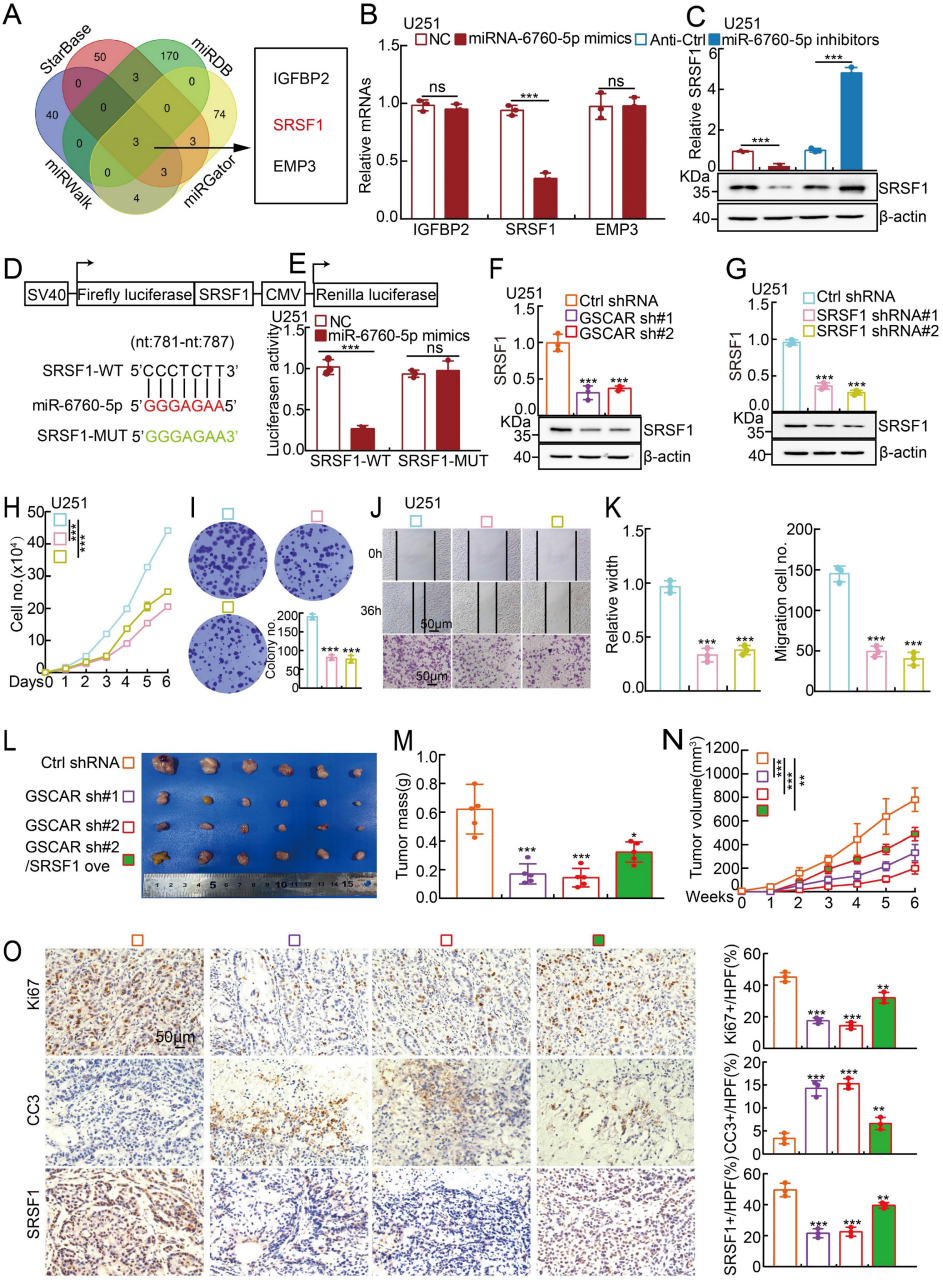
** MiR-6760-5p inhibited the expression of the oncogene SRSF1 in gliomas. (A)** Identification of the direct downstream target of miR-6760-5p using different web-source datasets (StarBase, miRDB, miRGator, and miRWalk). **(B-C)** The relative expression of the indicated genes after overexpressing miR-6760-5p mimics or inhibitors in U251 cells was examined by RT-PCR (B) or immunoblotting (C). **(D)** A schematic picture of the wild-type (WT) and mutant (MUT) SRSF1 3'-UTR-containing luciferase reporter plasmids.** (E)** The luciferase activities of the SRSF1 3'-UTR containing luciferase reporters (WT or MUT) were examined in U251 cells with miR-6760-5p mimics or NC coexpression. **(F-G)** The relative expression of SRSF1 was examined by RT-PCR (top) and immunoblotting (bottom) after GSCAR (F) or SRSF1 (G) knockdown. **(H-I)** SRSF1 knockdown markedly inhibited U251 cell proliferation, as shown by growth curve (H) and colony formation assays (I). Quantification results are indicated. **(J-K)** GSCAR knockdown inhibited cell migration in wound healing and transwell assays (J). Quantification results are indicated** (**K**)**. **(L-N)** GSCAR knockdown inhibited xenograft tumor formation *in vivo*. Representative xenograft tumor images (L), tumor masses (M), and tumor volumes (N) are shown. **(O)** Representative IHC staining of Ki67, cleaved caspase 3 (CC3), and SRSF1 in the indicated xenograft tumors. Quantification results are also indicated. Scale bar=50 μm. * *P* < 0.05, ** *P* < 0.01, *** P < 0.001. HPF=high power field, SRSF1 ove=SRSF1 overexpression.

It has been documented that miRNAs inhibit the targeted mRNAs 30. We then decided to identify the miR-6760-5p downstream target(s) using the StarBase, miRDB, miRWalk and miRGator datasets 32, 36-38, and IGFBP2, SRSF1, and EMP3 were identified **(Figure 4A).** To validate the specific target, miR-6760-5p mimics and inhibitors were introduced into glioma cells, respectively, and the transcripts of IGFBP2, SRSF1, and EMP3 were examined by RT-PCR. We revealed that only SRSF1, but not IGFBP2 and EMP3, was markedly reduced by miR-6760-5p mimics but increased by miR-6760-5p inhibitors in glioma tumor cells, suggesting that SRSF1 is the direct target of miR-6760-5p **(Figure 4B-4C, Figure S4A-S4B and Table S6)**. Previous studies have shown that SRSF1, predominantly driven by Myc, is highly expressed in multiple types of human cancer, including gliomas, and serves as an oncogenic factor via oncogenic splice-switching of MYO1B 39, 40. ROC curve analysis of SRSF1 showed an AUC value of 0.784 **(Figure S4C)**. We then verified the miR-6760-5p/SRSF1 axis using dual-luciferase reporters expressing either the wild-type (WT) or mutant 3'-UTR of the SRSF1 transcript **(Figure 4D-4E and Figure S4D)**. The high expression of SRSF1 in glioma patients correlated with adverse unfavorable clinical features** (Figure S4E-4F)**. In line with the former findings, we uncovered that miR-6760-5p expression was negatively associated with SRSF1 expression, while GSCAR expression was positively associated with SRSF1 expression in gliomas **(Figure S4G)**. Furthermore, we showed that GSCAR knockdown decreased SRSF1 expression, as examined by RT-PCR and immunoblotting **(Figure 4F and Figure S4H)**. In addition, we confirmed that SRSF1 knockdown inhibited glioma cell growth and migration and showed that SRSF1 overexpression could reverse GSCAR knockdown-reduced cell growth and migration abilities **(Figure 4G-4K and Figure S4I-4O)**.

To investigate the *in vivo* function of the GSCAR/miR-6760-5p/SRSF1 axis, a xenograft tumor formation assay was performed. Male nude mice at 5 weeks of age were randomly divided into 4 groups, and U251 cells stably expressing control shRNA or GSCAR-targeting shRNAs with or without SRSF1 forced-expressing vector were inoculated subcutaneously (1x10^6^ cells/point). In line with previous findings *in vitro*, GSCAR knockdown markedly inhibited tumor growth *in vivo*, as evidenced by the decreased Ki67 and increased cleaved caspase 3 (CC3) immunohistochemical (IHC) staining signals, which could be overcome by SRSF1 overexpression **(Figure 4L-4O)**.

### GSCAR promotes GSC maintenance

**Figure 5:**
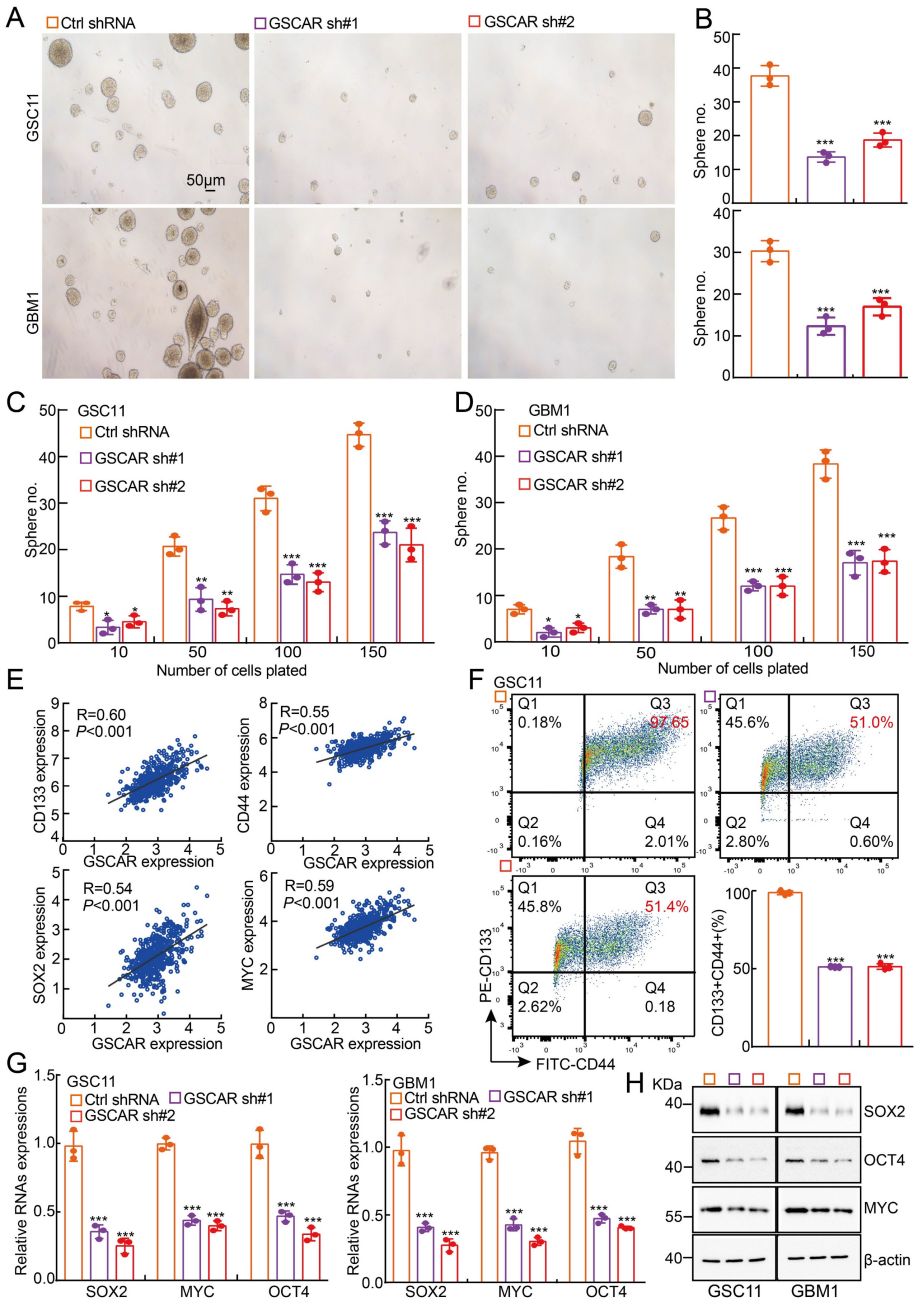
** GSCAR promoted stemness maintenance in glioma stem cells. (A-B)** Representative images of GSC11 and GBM1 tumor spheres expressing GSCAR shRNAs or control shRNA are shown. Scale bar: 50 μm. (B) Quantification data for (A). **(C-D)** *In vitro* limiting dilution assay for GSC11 (C) and GBM1 (D) cells expressing GSCAR shRNAs or Ctrl shRNA, respectively. **(E)** The positive correlations between GSCAR and stem cell maintenance-related genes, including CD133, CD44, SOX2, and c-MYC, were examined using the TCGA-LGG dataset by Pearson's correlation analysis. **(F)** Indicated cells were stained with a PE-labeled anti-AC133 (130-113-186, Miltenyi Biotec) and a FITC-labeled anti-CD44 antibody followed by flow cytometry analysis (n=3). Quantification results are indicated. **(G-H)** Knockdown of GSCAR inhibited GSC marker gene expression in GSCs, as verified by RT-PCR (G) and immunoblotting (H). The indicated antibodies were used. * *P* < 0.05, ** P < 0.01, *** *P* < 0.001.

Consistent with our bioinformatics analysis showing that GSCAR is highly expressed in glioma stem cells and TMZ-resistant cells **(Figure 1A and 1H)**, GSCAR is robustly increased in tumorsphere culture-enriched cells compared to parental adherent cells 7 **(Figure S5A)**. The existence of glioma stem cells is one of the major reasons for therapeutic resistance or cancer relapse 7, 41. We then decided to examine whether GSCAR regulates GSC maintenance and found that GSCAR knockdown indeed inhibited GSC11 and GBM1 self-renewal ability, as examined by tumorsphere culture and limiting dilution assays **(Figure 5A-5D)**. As expected, GSCAR expression positively correlated with glioma stem cell biomarkers, including CD133, CD44, NANOG, ALDH1, Oct4, SOX2, and c-Myc **(Figure 5E and Figure S5B)**. Knockdown of GSCAR markedly reduced the cell membrane accumulation of CD133+/CD44+ cells, as examined by FACS analysis, as well as SOX2, Oct4, and c-Myc expression, as detected by RT-PCR and immunoblotting, suggesting that GSCAR plays a critical role in GSCs **(Figure 5F-5H and Figure S5C)**. However, miR-6760-5p or SRSF1 was not increased in GSC-like cells compared to parental cells and was also not positively associated with the expression of glioma stem cell biomarkers **(Figure S5D-5F)**. Furthermore, knockdown of SRSF1 or overexpression of miR-6760-5p mimics did not affect tumor sphere formation in GSC11 or GBM1 cells **(Figure S5G-5H)**. The above results indicated that GSCAR promotes GSC self-renewal ability independent of the miR-6760-5p/SRSF1 axis.

### GSCAR/DHX9-IGF2BP2 complex activates GSCs by stabilizing SOX2 mRNA

**Figure 6:**
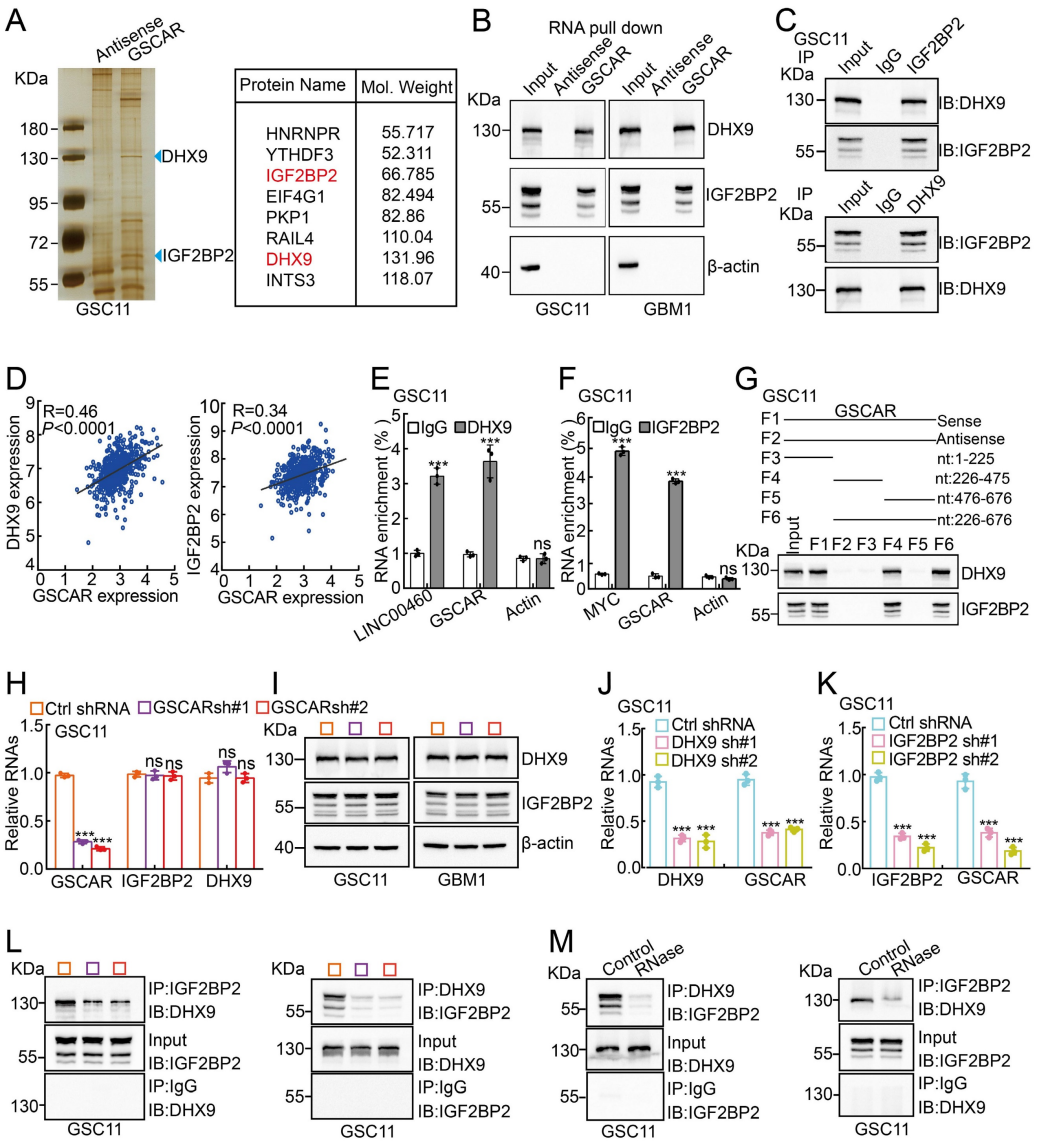
** GSCAR facilitated the interaction between DHX9 and IGF2BP2. (A)** Silver staining for biotinylated GSCAR-interacting proteins was followed by mass spectrometry analysis. Blue arrowheads indicate the candidate differentially identified proteins. **(B)** The interactions between GSCAR, DHX9, and IGF2BP2 were verified by RNA pull-down assay followed by immunoblot detection of the indicated proteins in GSC11 and GBM1 cells. Biotin-labeled GSCAR and its antisense RNA were transcribed *in vitro* using T7 RNA polymerase. β-Actin was used as a negative control. **(C)** The interaction between DHX9 and IGF2BP2 was examined by immunoprecipitation assay in GSC11 cells. **(D)** The correlations between DHX9/IGF2BP2 and GSCAR expression were examined using the TCGA-LGG dataset with Pearson's correlation analysis. **(E-F)** The protein-RNA interaction was verified by RIP assay in GSC11 cells. LINC00460, β-actin, or c-MYC was used as reciprocal control. **(G)** The RNA-protein interaction fragments were identified using serial truncated forms of GSCAR by RNA pull-down assay. Immunoblotting was performed using the indicated antibodies. **(H-I)** Effect of GSCAR knockdown on the expression of IGF2BP2 and DHX9 in GSC11 cells, as assessed by RT-PCR and immunoblot assays. **(J-K)** The relative RNA expression levels of the indicated genes were examined by RT-PCR. **(L-M)** Depleting GSCAR by shRNA (L) or RNase treatment (M) markedly reduced the interaction between DHX9 and IGF2BP2. * *P* < 0.05, ** P < 0.01, *** *P* < 0.001.

To decipher the mechanism by which GSCAR regulates GSC maintenance, we decided to validate GSCAR-interacting proteins using an RNA pull-down assay 42, and antisense GSCAR RNA was used as a negative control **(Table S3)**. The specific interactome of GSCAR was identified by SDS-PAGE followed by mass spectrometry (MS) analysis in GSC11 cells, and most of the candidates belonged predominantly to a class of RNA processing proteins **(Figure 6A and Figure S6A)**. To validate the MS candidates, we performed an immunoblot assay using the RNA pull-down materials in GSC11 and GBM1 cells **(Figure 6B)**. Among the candidate interacting partners, DHX9 and IGF2BP2 caught our attention for the following reasons: 1) the specificity of GSCAR binding; 2) the molecular weights for the most significant differentially detected proteins in SDS‒PAGE; and 3) consistent with a previous study, we showed that IGF2BP2 and DHX9 interact with each other 43, which were increased in gliomas and positively correlated with GSCAR expression and adverse clinical outcomes **(Figure 6C-6D and Figure S6B-6D)**. Additionally, we performed RIP assays to validate the specificity of the GSCAR-IGF2BP2/DHX9 interactions, and LINC00460 or Myc was used as a reciprocal control **(Figure 6E-6F and Figure S6E-6F)** 44, 45. To identify the interacting region in GSCAR mediating DHX9/IGF2BP2 binding, 4 biotinylated GSCAR fragments, including nt 1-225, nt 226-475, nt 476-676, and nt 226-676, were constructed and used for the RNA pull-down assay, and only the fragment containing nt 226 to 475 could interact with DHX9 or IGF2BP2 **(Figure 6G).** In addition, the mRNA and protein expression levels of DHX9 or IGF2BP2 were not deregulated upon GSCAR knockdown, while knockdown of DHX9 or IGF2BP2 decreased GSCAR expression in GSCs but not in U251 cells **(Figure 6H-6K and Figure S6G-6K)**. Importantly, we detected that GSCAR knockdown or RNase treatment markedly reduced the interaction between IGF2BP2 and DHX9, indicating that GSCAR is critical for DHX9/IGF2BP2 complex formation** (Figure 6L-6 M)**.

**Figure 7:**
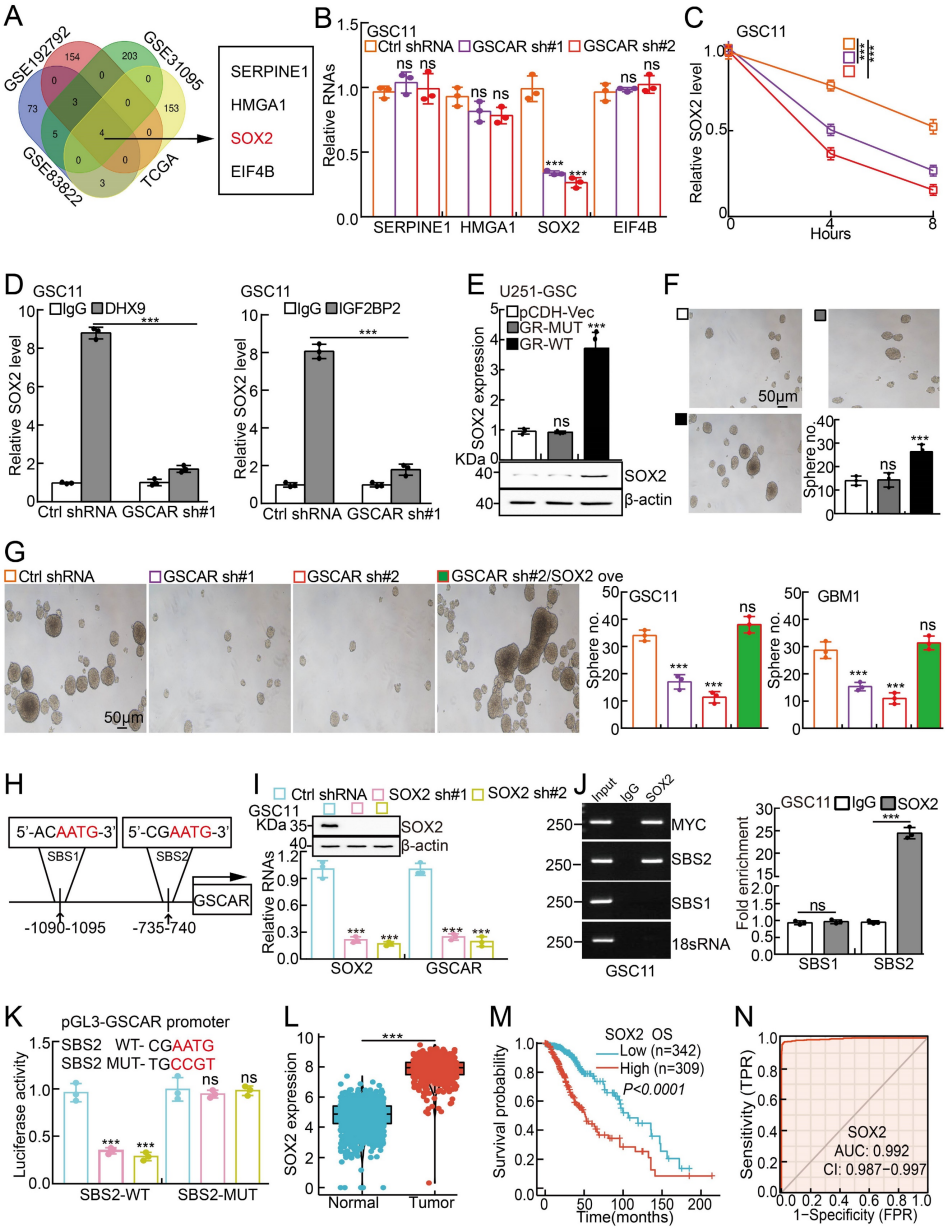
** The GSCAR/DHX9-IGF2BP2 complex stabilized SOX2 mRNA. (A)** SOX2 was identified by integrative omics analysis using GEO datasets, GSE192792 (blue): data generated from CLIP-seq analysis of IGF2BP2 binding targets in glioma cells, GSE31095 (red): data generated from glioma tissue samples (logFC>3, *P*<0.001), GSE83822 (green): data generated from CLIP-seq analysis of DHX9 binding targets in glioma cells, and TCGA (yellow): glioma TCGA dataset (R>0.4, *P*<0.001). **(B)** The relative expression of the indicated genes after GSCAR knockdown was examined by RT-PCR in GSC11 cells. **(C)** The decay rate of SOX2 mRNA after actinomycin D (5 μg/ml) treatment in the indicated cells. **(D)** The protein‒RNA interaction was verified by RIP assay in GSC11 cells after GSCAR knockdown compared to the control shRNA group. **(E-F)** Forced expression of wild-type GSCAR but not the GSCAR mutant lacking nt 226 to 475 in U251 cells increased SOX2 expression, as examined by RT-PCR and immunoblotting (E), and tumorsphere formation ability (F). Quantification results are also indicated. Scale bar: 50 μm. **(G)** Tumorsphere formation assay in the indicated cells. Quantification results are also indicated. Scale bar: 50 μm. **(H)** The potential SOX2 binding site (SBS) in the promoter region of GSCAR is shown. **(I)** SOX2 knockdown-inhibited GSCAR expression in GSC11 cells was examined by RT-PCR. Immunoblotting was performed to verify SOX2 knockdown efficiency. **(J)** SOX2 bound to the SBS2 site verified by ChIP assay. Quantification results are also indicated. **(K)** SOX2 transcriptionally induced GSCAR expression by the SBS2 site, as examined by dual-luciferase reporter assay. **(L)** The relative expression level of SOX2 in TCGA and GTEx datasets (Normal:1152, Tumor:523).** (M)** Kaplan-Meier analysis of the overall survival curve of glioma patients with different SOX2 expression levels. **(N)** The ROC curve for SOX2 (AUC=0.992) in gliomas using the TCGA dataset. * *P* < 0.05, ** P < 0.01, *** *P* < 0.001. pCDH-Vec=pCDH lentiviral plasmid vector control. GR-MUT=PCDH-GSCAR MUT, GR-WT=PCDH-GSCAR WT, GSCAR sh#1=GSCAR shRNA#1.

Previous findings have demonstrated that DHX9 and IGF2BP2 can form a complex to regulate the stabilities of multiple interacting mRNAs 43, 46. To explore the direct downstream mRNA regulated by the GSCAR/IGF2BP2-DHX9 complex in GSCs, we applied multiple web-source available datasets and identified high mobility group A1 (HMGA1), SERPINE1, eukaryotic initiation factor 4B (EIF4B), and SOX2 as the common interacting mRNAs **(Figure 7A and Table S7)**. However, we observed that only SOX2, but not HMGA1, SERPINE1, or EIF4B transcripts, was markedly reduced after GSCAR knockdown **(Figure 7B and Figure S7A)**. Moreover, the SOX2 mRNA decay rate was examined, which was dramatically increased after GSCAR knockdown compared to the control group **(Figure 7C and Figure S7B)**. Consistently, we showed that the endogenous DHX9 or IGF2BP2 proteins bound to SOX2 mRNA by protein-RNA immunoprecipitation assay, which was markedly reduced upon GSCAR knockdown **(Figure 7D and Figure S7C)**. In line with this finding, forced expression of GSCAR but not the GSCAR mutant lacking the nt 226 to 475 fragment in U251 or A172 cells increased SOX2 expression and the stem-like phenotype in the tumorsphere culture assay **(Figure 7E-7F and Figure S7D-7E)**. Consistent with former findings that SOX2 promote Myc and Oct4 expressions 47, 48, we found that SOX2 knockdown reduced GSCAR overexpression increased self-renewal ability in GSCs **(Figure S7F)**. In addition, SOX2 overexpression could reverse the reduced sphere formation ability and Myc/Oct4 expressions upon GSCAR knockdown in GSCs **(Figure 7G and Figure S7G-7H)**. Knockdown of IGF2BP2 or DHX9 significantly inhibited GSCAR overexpression-induced SOX2 upregulation in GSCs **(Figure S7I).** The above results indicate that GSCAR is essential for the IGF2BP2 and DHX9 interaction and GSC stemness maintenance in a SOX2-dependent manner. Interestingly, we detected 2 potential SOX2 binding sites (SBS1 and SBS2) within the GSCAR promoter region 49, 50, and SBS2 but not SBS1 was confirmed to be the direct binding site mediating SOX2 transcriptional induction by dual-luciferase reporter and chromatin immunoprecipitation assays **(Figure 7H-7K)**. Consistent with former findings 51, SOX2 was verified to be highly expressed in gliomas, which correlates with worse clinical outcomes **(Figure 7L-7N)**. These results suggest that the GSCAR/DHX9-IGF2BP2 complex and SOX2 form a positive feedback loop to regulate GSC maintenance.

### GSCAR/DHX9-IGF2BP2/SOX2 feedback loop is critical for glioma progression

**Figure 8:**
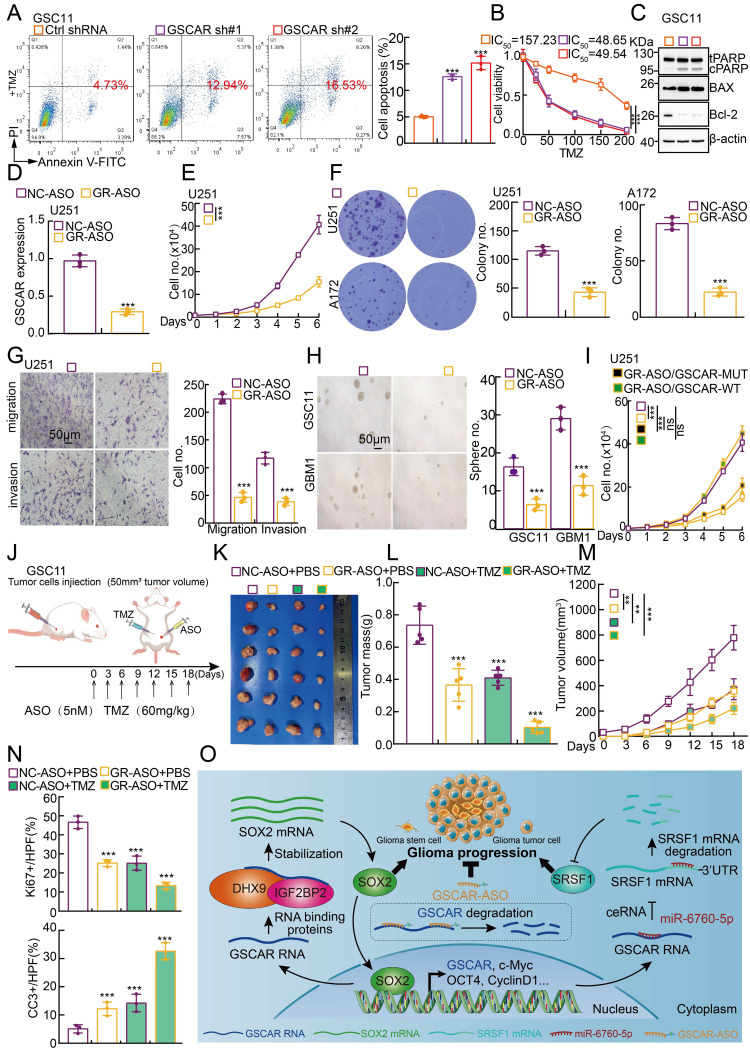
** GSCAR-targeting ASO impeded tumor growth. (A-B)** GSCAR knockdown promoted TMZ-induced cellular apoptosis in GSC11 cells as detected by flow cytometry (A) and SRB (B) assays. Quantification results are indicated. **(C)** Marker genes related to cellular apoptosis were detected by immunoblotting with the indicated antibodies.** (D)** The relative expression of GSCAR was examined by RT -PCR after transfection with the indicated ASO. **(E-F)** GSCAR-targeting ASO inhibited cell proliferation, as examined by growth curve (E) and colony formation (F) assays. Quantification results are indicated. **(G)** GSCAR-targeting ASO repressed cell migration and invasion in the transwell assay. Scale bar=50 μm. **(H)** GSCAR-targeting ASO inhibited cancer stem cell self-renewal ability by tumorsphere formation assay. Quantification results are indicated. Scale bar=50 μm. **(I)** GSCAR wild-type but not mutant was able to rescue GSCAR-targeting ASO reduced cell growth phenotype. GSCAR-WT=GSCAR wild-type; GSCAR-MUT=GSCAR mutant=GSCAR targeting ASO insensitive mutant. **(J)** Schematic view of the xenograft mouse model treated with the indicated ASO (5 nM) and TMZ (60 mg/kg). **(K-M)** Representative xenograft tumor images (K), tumor masses (L), and tumor volumes (M) are shown for the indicated groups treated with the indicated ASO with or without TMZ. GSC11 cells were used. **(N)** Quantified results for the IHC staining of Ki67 and CC3 are presented in the indicated xenograft tumor sections. **(O)** Schematic diagram showing how GSCAR promotes glioma progression via both the GSCAR/miR-6760-5p/SRSF1 and GSCAR/DHX9-IGF2BP2/SOX2 axes. * *P* < 0.05, ** P < 0.01, *** *P* < 0.001. NC-ASO = ASO negative control; GR-ASO=GSCAR targeting ASO.

Based on the fact that GSCs are important for chemotherapy resistance in glioma 7, 41, we decided to verify whether blocking the GSCAR/DHX9-IGF2BP2/SOX2 feedback loop could increase the glioma cell response to TMZ. As expected, increased cellular apoptosis was detected upon GSCAR knockdown compared to the control group **(Figure 8A-8B and Figure S8A-8B)**, which was further verified by immunoblot detecting the expression of PARP, Bax, and Bcl-2 **(Figure 8C and Figure S8C)**. Antisense oligonucleotide (ASO) drugs have recently been developed to inhibit tumor growth 30. The high expression of GSCAR in gliomas and its critical role in promoting tumor progression prompted us to exploit the potential of GSCAR as a therapeutic target by using ASO. Therefore, one independent ASO specifically targeting GSCAR and the control ASO were designed and transfected into glioma tumor cell lines. The cell growth, migration and tumorsphere formation abilities of glioma tumor cells were examined and were unanimously decreased by GSCAR-targeting ASOs compared to the control group** (Figure 8D-8H and Figure S8D-8F)**. In addition, we showed that GSCAR wild-type but not mutant was able to rescue GSCAR-targeting ASO reduced cell growth phenotype **(Figure 8I and Figure S8G)**. In line with the *in vitro* findings, the antitumor efficacy of GSCAR-targeting ASOs was examined using a xenograft tumor model. Five-week-old male nude mice were randomly divided into 4 groups, and 1 X 10^6^ GSC11 cells were subcutaneously injected. The control ASO and GSCAR-targeting ASO were injected around tumors with or without TMZ until the tumor volume reached 50 mm^3^ according to the schematic cartoon **(Figure 8J)**. We found that GSCAR-ASO dramatically inhibited tumor growth *in vivo* alone or in combination with TMZ compared to the control group, as evidenced by the reduced tumor masses and volumes **(Figure 8K-8M)**. Consistently, the IHC staining of Ki67, CD133, SOX2, and CD44 was significantly reduced, while cleaved caspase 3 (CC3) staining was increased, in GSCAR-ASO with or without TMZ-treated xenograft tumors compared to the reciprocal control groups **(Figure 8N and Figure S8H-8J)**. In conclusion, GSCAR might serve as a therapeutic target in gliomas in the future.

## Discussion

Surgical resection, radiotherapy and chemotherapy are currently the standard treatment options for glioma patients, while limiting effects have been achieved 52. Therefore, deciphering the molecular mechanisms of TMZ resistance or identifying combined therapeutic strategies would greatly benefit clinical outcomes. Recent studies have shown that lncRNAs play important roles in the malignant process of gliomas 53-55. Here, we identified a human brain-specific long noncoding RNA GSCAR that was highly expressed in glioma cancerous tissues and cell lines due to aberrant somatic copy number alterations. Increased GSCAR expression correlated with poor clinical outcomes in glioma patients. We showed that GSCAR interacted with miR-6760-5p to increase the expression of the oncoprotein SRSF1, leading to increased tumor growth and migration in U251 and A172 cells. In this study, we showed that miR-6760-5p was decreased in glioma cells, and overexpression of miR-6760-5p inhibited cell proliferation and migration abilities. Rescue experiments showed that GSCAR overexpression could partly reverse the reduced cell proliferation and migration abilities induced by miR-6760-5p mimics overexpression. These results indicate that additional mechanisms mediated by miR-6760-5p may exist regulating tumor cell growth and migration. In line with our findings, previous studies have shown that SRSF1 was increased in gliomas, which promoted gliomagenesis via guided alternative splicing of the MYO1B gene, leading to activation of PDK1/AKT and PAK/LIMK signaling pathways 39. Interestingly, GSCAR was identified to increase the stemness of glioma stem cells independent of the GSCAR/miR-6760-5p/SRSF1 axis.

The mechanistic study showed that GSCAR mediated the interaction between IGF2BP2 and DHX9. IGF2BP2 has been identified as a member of the RNA binding protein family (IGF2BP), which plays critical roles in RNA localization, translation, and stability 56. DHX9 play crucial roles in the gene transcription 57, RNA stability 58, translation 59, RNA processing and transport 60. One recent study found that lncRNA HIF1A-AS2 interacted with IGF2BP2 and DHX9 to stabilize HMGA1 mRNA 46, and HMGA1 has been shown to promote glioma malignant progression by activting the PI3K/Akt/c-Jun signaling pathway 61. However, we did not observe that HMGA1 mRNA was significantly reduced after GSCAR knockdown in GSCs, suggesting that HMGA1 could be regulated either by IGF2BP2 or DHX9 in glioma tumor cells in different cellular contexts.

Here, we provided evidence showing that the SOX2 transcript was markedly reduced after GSCAR knockdown in GSCs, resulting from the loss of interaction between but not deregulated expression of IGF2BP2 and DHX9, indicating that the GSCAR/DHX9-IGF2BP2 complex specifically stabilizes SOX2 mRNA in GSCs. SOX2 has been shown to be essential for stem cell self-renewal and homeostasis 62, 63, and inhibit cell growth by inducing apoptosis in different types of human cancer 64, 65. In addition, we revealed that SOX2 activated GSCAR expression at the transcriptional level in GSCs. Numerous lncRNAs have been reported to be located in the cytoplasm and play pivotal biological functions, including serving as microRNA (miRNA) sponges, interacting with RBPs, and even translating proteins 66. The positive feedback loop of GSCAR/DHX9-IGF2BP2/SOX2 distinguishes glioma stem cells from other glioma tumor cells regulated by the GSCAR/miR-6760-5p/ SRSF1 axis **(Figure 8O)**.

Based on the critical role of SOX2 in cancer stem cell maintenance and cancer progression, it has become an effective therapeutic target, and SOX2 peptide immunization has been shown to delay tumor onset and activate cytotoxic T lymphocytes in mouse models 67. However, given the fundamental role of SOX2 in normal stem cell homeostasis and developmental processes, targeting SOX2-related signaling pathways might be more desirable than directly blocking SOX2 to avoid severe side effects. Recently, targeting noncoding RNAs (ncRNAs) has become a promising therapeutic option in human cancers. Small molecules targeting oncogenic ncRNA can selectively result in the apoptosis of cancerous cells 68, 69. Moreover, our group reported that ASO targeting lncRNA PKMYT1AR significantly inhibited NSCLC progression 30. The blood-brain barrier is the most restrictive barrier preventing most drugs from entering the brain in glioma patients, while Lei Dong et al. found that ASOs can be efficiently delivered across the BBB by tumor cell-derived small apoptotic bodies 70. Our results showed that GSCAR-targeting ASOs alone or in combination with TMZ markedly inhibited glioma tumor cell growth both *in vitro* and *in vivo*, suggesting that GSCAR is a promising therapeutic target for glioma patients in the future.

## Conclusions

Our results reveal that the GSCAR/miR-6760-5p/SRSF1 axis is important for tumor growth, while the GSCAR/DHX9-IGF2BP2/Sox2 feedback loop is critical for GSC maintenance and TMZ resistance. Blocking GSCAR expression efficiently inhibits glioma progression, indicating that GSCAR and its related molecular events could be used as novel therapeutic targets for gliomas in the future.

## Supplementary Material

Supplementary methods, figures and tables.

## Abbreviations

GSCs: Glioma stem cells
TMZ: Temozolomide
GSCAR: Glioma stem cell-associated lncRNA
ASO: Antisense oligonucleotide
ceRNA: competing endogenous RNA
SRSF1: Serine and arginine rich splicing factor 1
DHX9: DExH-Box helicase 9
IGF2BP2: Insulin-like growth factor 2 mRNA-binding protein 2
SOX2: Sex-determining region Y-box 2
GBM: Glioblastoma
LGG: Low grade gliomas
NSPC: Neural stem and progenitor cell
HOTAIR: HOX transcript antisense RNA
HGG: High-grade gliomas
SCNA: Somatic copy number alterations
WT: Wild-type
MUT: Mutant
ISH assay: RNA *in situ* hybridization
ROC: Receiver operating characteristic curve
AUC: Area under the curve
NHA: Fetal normal human astrocyte
FISH: RNA fluorescence *in situ* hybridization
KEGG: Kyoto Encyclopedia of Genes and Genomes
CC3: Cleaved caspase 3
IHC: Immunohistochemical
HMGA: High mobility group A1
SERPINE1: Serine protease inhibitor family E member 1
EIF4B: Eukaryotic initiation factor 4B
ChIP: Chromatin immunoprecipitation
RIP: RNA immunoprecipitation
GEO: Gene Expression Omnibus
TCGA: The Cancer Genome Atlas
GTE: Genotype-Tissue Expression
