# Supplementary material for: LncRNA GSCAR promotes glioma stem cell maintenance via stabilizing SOX2 expression
Source: Int J Biol Sci. 2023 Mar 5;19(6):1681–97. doi: 10.7150/ijbs.80873 (PMC10092759; doi:10.7150/ijbs.80873)
Supplement: Supplementary file 1 — Supplementary methods, figures and tables. [file ijbsv19p1681s1.pdf]

Supplementary information For

LncRNA GSCAR promotes glioma stem cell maintenance via stabilizing SOX2 expression

Xiulin Jiang[1, 2, 3 ‡], Yong Zhang[4, ‡], Yixiao Yuan[5, ‡], Zhixian Jin[5, ‡], Haoqing Zhai[3], Baiyang Liu[3], Yao Li[3], Chun Zhang[3], Min Chen[5], Yulin Shi[5], Dongming Yan[6*], Jun Pu[5,*], Yongbin Chen[3, *], Cuiping Yang[1, 2, 3*]

‡ These authors contributed equally to this work.

[*] Correspondence email: Cuiping Yang: cuipingyang@sjtu.edu.cn

Yongbin Chen: ybchen@mail.kiz.ac.cn

Jun Pu: pujun303@aliyun.com

Dongming Yan: mrdmyan@163.com

This PDF file includes:

1、Additional Methods

2、Supplementary Figures. S1 to S8

3、Supplementary Tables. S1 to S7

Additional Methods

Cell culture, cell proliferation and colony formation

The control and glioma cell lines were cultured at 37˚C in a 5% CO2 humidified environment. Briefly, NHA cells were cultured using the commercial astrocyte medium (catalog 1801, ScienCell) supplemented with 2% FBS (ScienCell), 1% AGS (ScienCell), and 1% penicillin/streptomycin (ScienCell). U251, U87, A172, and HEK-293T cells were all cultured in DMEM medium (Hyclone) supplemented with 10% fetal bovine serum (FBS, Gibco, 10270-106) and 1% penicillin/streptomycin. The GSC11 cells were cultured in serum-free DMEM/F12, supplemented with B27 (1:50, Invitrogen, 2175161), 20 ng/mL EGF (Gbico, PHG0311L), 20 ng/mL bFGF (Gbico, PHG0266), 4 ng/mL heparin (Sigma, H3149-500KU-9) and 1% penicillin/streptomycin. The GBM1 and GBM2 were cultured in neurobasal A medium (Gibco, 2085380) supplemented with B27, GlutaMAX (1:100, Gibco 35-50-061), Sodium pyruvate (1:100, Gibco R25-0000-CI), MEM NEAA (1:100, Gibco, 11140-050), 20 ng/mL bFGF and 1% penicillin/streptomycin. The cell proliferation, colony formation, and BrdU incorporation assays were performed. Briefly, for cell proliferation assay, indicated cells were plated into 12-well plates and the cell numbers were subsequently counted each day using an automatic cell analyzer countstar (Shanghai Ruiyu Biotech Co., China, IC 1000). For colony formation assay, indicated cells were seeded in 6-well plates (China, NEST, Cat. 703001), and the cell culture medium was changed every 3 days for 2~3 weeks, and then indicated cells were fixed with 4% PFA and stained with 0.5% crystal violet.

Cell migration and invasion assays

To produce a wound, the monolayer cells in 6-well plates were scraped in a straight line with pipette tips. Plates were then washed with PBS to remove detached cells. Images of the scratches were taken at indicated time points using Nikon inverted microscope (Ti-S). The relative gap width was calculated using GraphPad Prism software. For transwell assay, indicated cells in 100μL serum-free medium were plated in a 24-well plate chamber insert (Corning Life Sciences, Cat. 3422), with the medium containing 10% FBS at the bottom of the insert. For invasion assay, the upper chamber of the insert was pre-coated with Matrigel (Millipore Sigma) before plating cells. After incubation for 48 h, cells were fixed with 4% paraformaldehyde for 1 hour and then stained with 0.1% crystal violet for 30 min. After rinsing with water, migrating or invading cells were imaged and quantified.

Cell flow cytometry assays

For cell apoptosis detection, Annexin V FITC Apoptosis Detection Kit I (556547, BD, China) was used to evaluate the cellular apoptosis following the manufacturer's instructions. For cell cycle analysis, indicated cells were digested and washed with PBS twice and then fixed in 75% alcohol overnight at - 20 °C. The fixed cells were washed and then stained with propidium iodide (PI) staining buffer. Indicated cells were then analyzed by the FACSAria SORP machine (BD, USA).

RNA subcellular fractionation and fluorescence in situ hybridization assay

The nuclear and cytoplasmic fractions were isolated using the NORGEN kit (Cat. 21000, NORGEN, USA). The indicated cells were lysed using cell fraction buffer on ice for 10 min, and after centrifugation at 5000 g for 5 min at 4 °C, the supernatant or the pellet was collected for further cytoplasmic or nuclear fraction purification, respectively. For the RNA fluorescence in situ hybridization (FISH) assay, a Cy3-labeled GSCAR probe was designed and synthesized by RiboBio (China), and the FISH kit (RiboBio,Kit, Cat. C10910) was used to determine the RNA expression pattern following the manufacturer's instructions.

Xenograft tumor formation assay

Indicated tumor cells were subcutaneously injected into 4-5 weeks old male nude mice (Purchased from Vital River Laboratories, Beijing). At the end of the experiments, all mice were sacrificed and the tumors were harvested, weighed, and photographed. Nude mice were monitored every day, the xenograft tumor volumes were measured with a sliding caliper, and tumor volumes were calculated using the formula $(L \times W^2)/2$. For TMZ and ASO treatment assay in vivo, until the xenograft tumors reached a volume of 50 mm^3, mice were randomly divided into indicated treatment groups. The nude mice were treated with ASOs (intratumoral injection) with or without TMZ (intraperitoneal injection) every 3 days, PBS intratumoral injection was used as the control group. All mice were sacrificed at the end of the experiment and tumors were harvested, pictured, and weighed. All animals were

kept in an SPF environment and the protocols were pre-approved and conducted under the policy of the Animal Care and Use Committee at the Kunming Institute of Zoology, CAS.

Dual-luciferase and RT-PCR assays

For the dual-luciferase assay, indicated DNA fragments were synthesized and cloned into pGL3 basic vector (**Table S1**), Renilla luciferase plasmid, and indicated plasmids were co-transfected into indicated cells, 24~48 hours later, the luciferase activities were examined by Dual Luciferase Kit (Promega, E1960). For the RT-PCR experiment, indicated RNAs were extracted by RNAiso Plus (Takara, 108-95-2), and then reverse transcribed by the PrimeScript RT reagent Kit (Takara Bio, RR047A). The cDNAs were used for RT-PCR assay using FastStart Universal SYBR Green Master Mix (Roche, 04194194001) and an Applied Biosystems 7500 machine. The detailed primer and oligo sequences used in this study were provided in **Table S1**.

Immunohistochemical staining (IHC)

Briefly, the tissue sections were deparaffinized in xylene and rehydrated using graded ethanol. Antigen retrieval was performed using sodium citrate buffer (pH 6.0), and the endogenous peroxidase activity was quenched with 3% H_2O_2, sections pretreated with 1% bovine serum albumin buffer were then incubated with indicated primary antibodies overnight at 4°C. After several washes, the sections were treated with HRP-conjugated secondary antibody for 40 min at room temperature and stained with

3, 3-diaminobenzidine tetrahydrochloride (DAB). Slides were photographed with a microscope (Olympus BX43F, Japan), and representative images were analyzed with the Image-Pro Plus 7.0 software (Media Cybernetics, Inc., Silver Spring, MD, USA).

Supplementary Figures and Figure legend

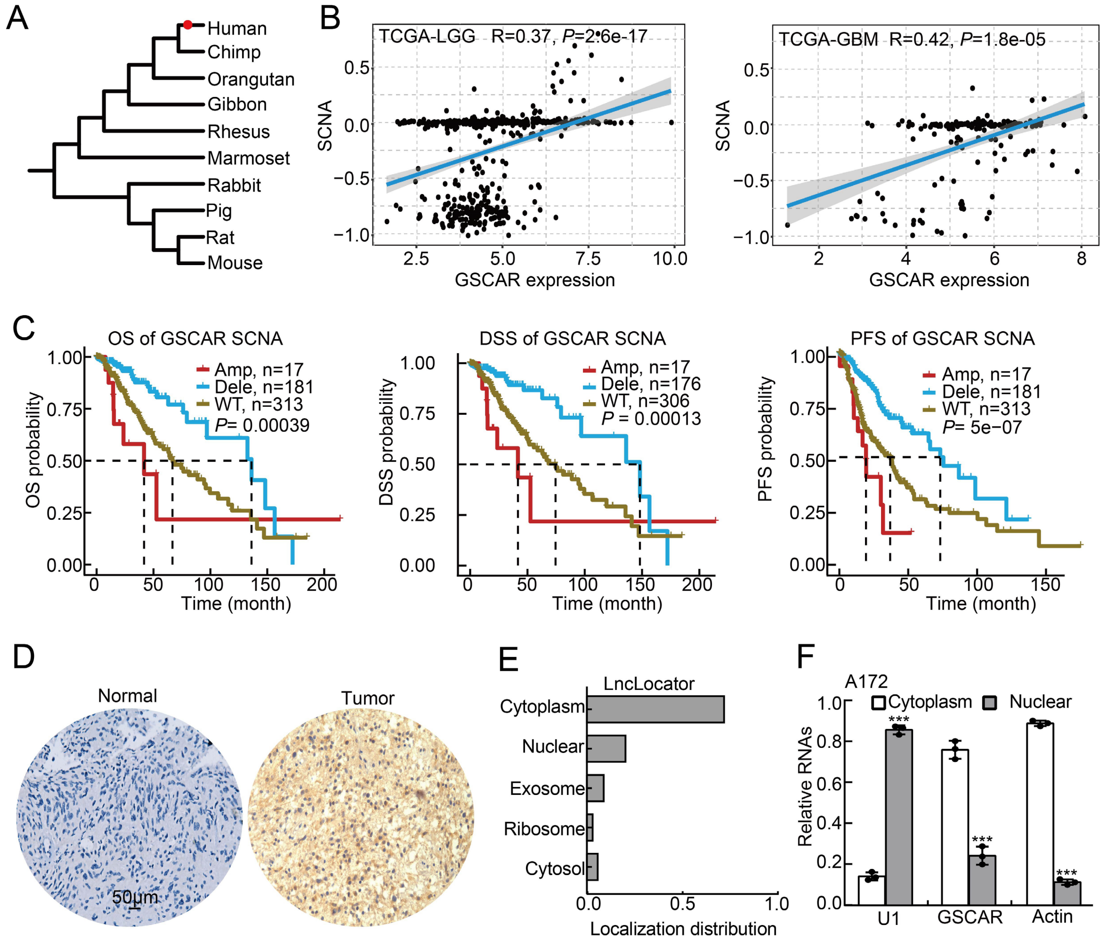

Figure S1 GSCAR was highly expressed in gliomas. (A) The sequence comparison of human-specific GSCAR was examined by Gentree (http://gentree.ioz.ac.cn/). **(B)** The correlation between GSCAR expression and the somatic copy number alterations (SCNAs) was examined by Gene Set Cancer Analysis (GSCA) database (http://bioinfo.life.hust.edu.cn). **(C)** The OS, DSS, and PFS of glioma patients with altered or unaltered GSCAR somatic copy number alterations (SCNAs) were examined by the GSCA database (http://bioinfo.life.hust.edu.cn/). Amp=Amplication, Dele=Deletion, WT=Wild Type. **(D)** The original tumor sections for figure 1f from

tissue microarray. **(E)** GSCAR was majorly localized in the cytoplasm examined by the Lnclocator database. **(F)** GSCAR was majorly localized in the cytoplasm of A172 cells using the nuclear and cytoplasmic RNA fractionation assay followed by the RT-PCR examination. β-actin (ACTB) and U1 were used as cytoplasmic and nuclear fraction controls, respectively. * $P < 0.05$, ** $P < 0.01$, *** $P < 0.001$.

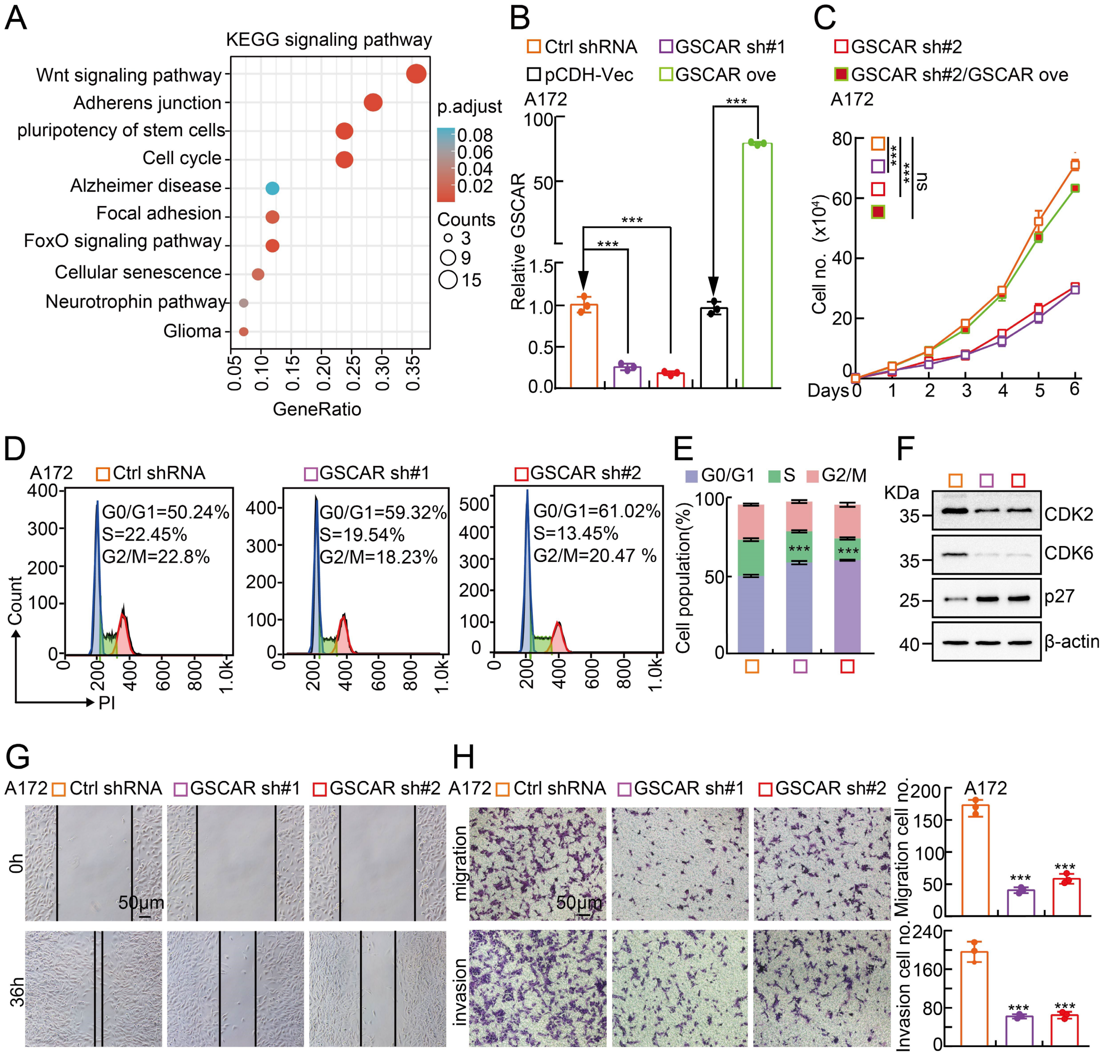

Figure S2 GSCAR knockdown inhibited glioma cell proliferation and migration.

(A) The Wnt signaling pathway, cell cycle, and focal adhesion signaling pathways were enriched by KEGG analysis. The detail data source was obtained from TCGA-LGG (Low grade gliomas, https://portal.gdc.cancer.gov/repository). **(B)** The establishment of GSCAR knockdown and overexpression cell lines in A172 was verified by RT-PCR. **(C)** GSCAR knockdown dramatically inhibited A172 cell proliferation examined by growth curve assay. **(D-E)** GSCAR knockdown blocked the G0/G1 cell cycle transition in A172 cells examined by PI staining and flow cytometry

assay. (E) Quantification data for (D). **(F)** GSCAR knockdown regulated the expressions of cell cycle transition mediators, including CDK2, CDK6 and p27. Indicated cell extracts were probed with indicated antibodies. **(G-H)** Knockdown of GSCAR inhibited A172 cell migration using wound healing (G) and transwell (H) assays. Scale bar=50 μm. Quantification results were also indicated. * $P < 0.05$, ** $P < 0.01$, *** $P < 0.001$.

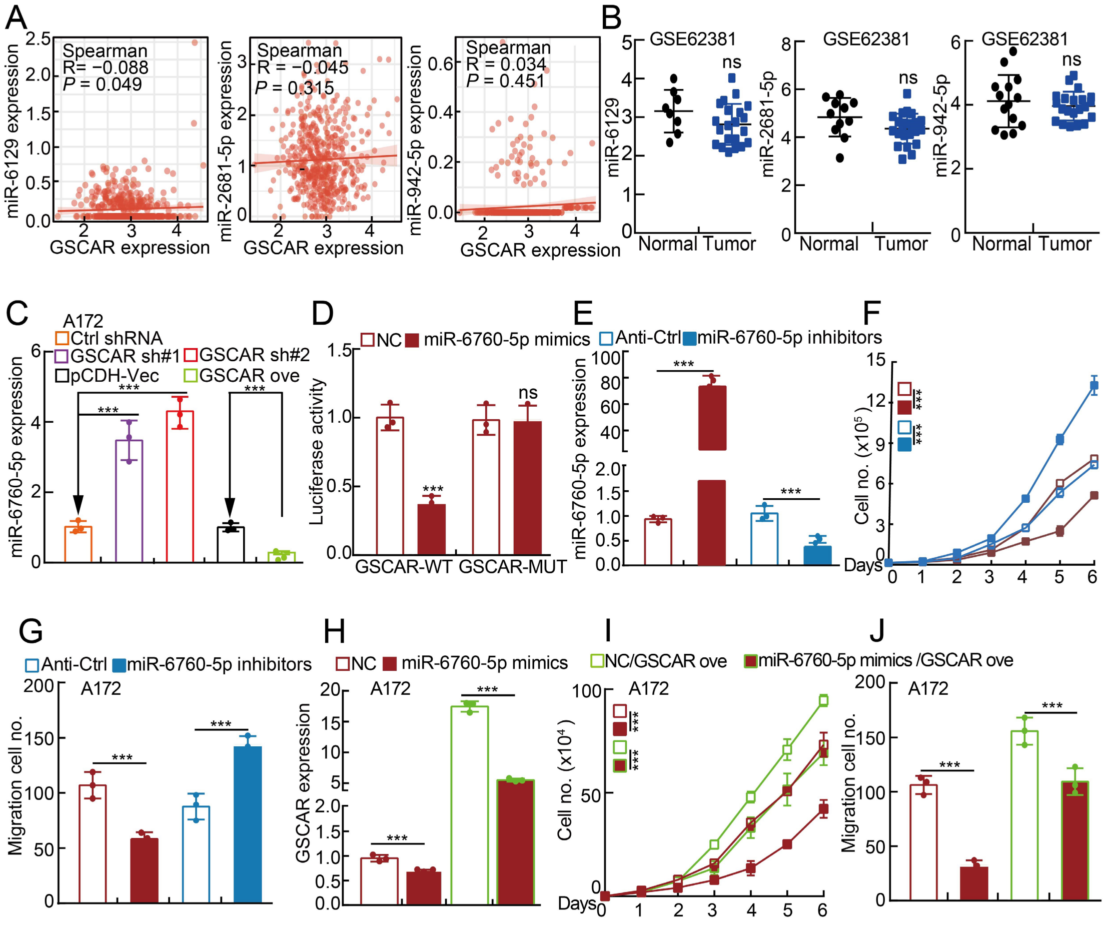

Figure S3 MiR-6760-5p inhibits glioma cell proliferation and migration. (A) Correlation analysis between GSCAR and miR-6129, miR-2681-5p, or miR-942-5p, respectively, using the TCGA-LGG dataset. **(B)** The relative expressions of miR-6129, miR-2681-5p, or miR-942-5p in gliomas. **(C)** The relative miR-6760-5p expression was examined by RT-PCR in indicated cells. **(D)** The luciferase activities of the GSCAR luciferase reporters (WT or MUT) were examined in A172 cells with miR-6760-5p mimics or NC co-expression. **(E)** The relative miR-6760-5p expression was examined by RT-PCR after indicated oligos transfection. **(F-G)** MiRNA-6760-5p mimics overexpression reduced while miRNA-6760-5p inhibitors overexpression promoted A172 cell growth (F) and migration (G). Quantification results were

indicated. **(H)** Relative GSCAR expression was examined by RT-PCR in the indicated cells. **(I-J)** GSCAR overexpression overcame the cell proliferation and migration abilities repressed by miR-6760-5p mimics overexpression. * $P < 0.05$, ** $P < 0.01$, *** $P < 0.001$.

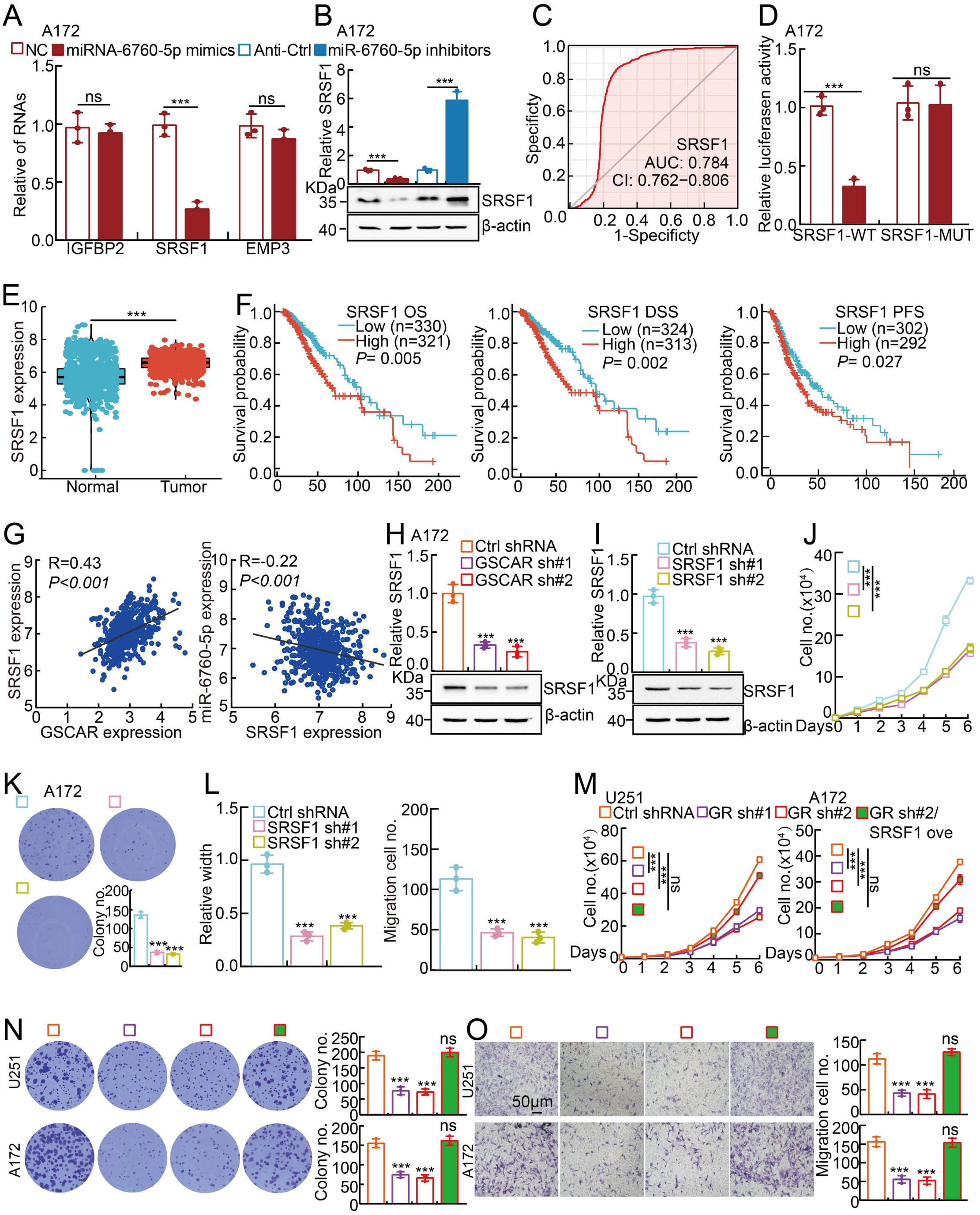

Figure S4 SRSF1 was targeted by miR-6760-5p in gliomas. (A) The relative expressions of indicated genes after overexpressing miR-6760-5p mimics or NC in A172 cells were examined by RT-PCR. **(B)** The relative expressions of SRSF1 were examined by RT-PCR (top) and immunoblot assay (bottom) in indicated cells. **(C)** The ROC curve for SRSF1 (AUC=0.784) in gliomas using the TCGA dataset. **(D)** The

luciferase activities of the SRSF1 3'-UTR containing luciferase reporters (WT or MUT) were examined in A172 cells with miR-6760-5p mimics or NC co-expression. **(E)** The expression level of SRSF1 in TCGA and GTEx datasets (Normal: 1152, Tumor: 523). **(F)** SRSF1 high expression correlated with OS, DSS, and PFS. **(G)** SRSF1 positively but negatively correlated with GSCAR and miR-6760-5p respectively examined by Pearson's correlation analysis using the TCGA-LGG dataset. **(H-I)** The relative SRSF1 expressions were examined by RT-PCR (top) and immunoblot (bottom) in indicated cells. **(J-L)** SRSF1 knockdown inhibited A172 cell proliferation, examined by growth curve (J) and colony formation (K) assays, while cell migration examined by wound healing and transwell assay (L). Quantification results were indicated. **(M-O)** Forced expression of SRSF1 reversed GSCAR knockdown-mediated phenotypes examined by growth curve (M), colony formation (N), and transwell (O) assays in indicated cells. Quantification results were also presented. * $P < 0.05$, ** $P < 0.01$, *** $P < 0.001$.

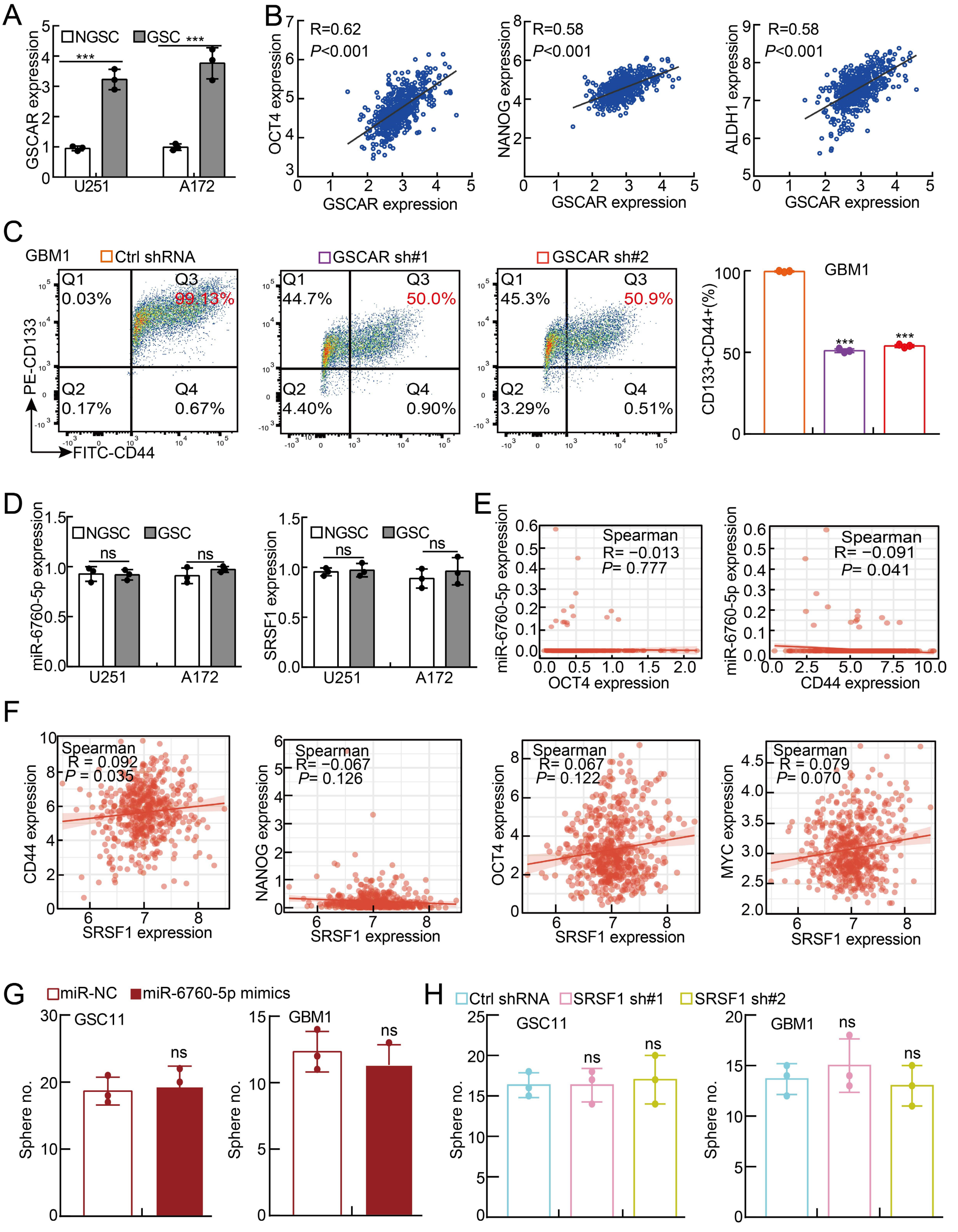

Figure S5 GSCAR regulated the stemness maintenance of GSCs. (A) The relative expression of GSCAR in spheroid- (GSC) and adherently- (NGSC) cultured U251 and A172 cells, respectively, were examined by RT-PCR. **(B)** The positive correlations between GSCAR and stem cell maintenance related genes, including

OCT4, NANOG, and ALDH1, were verified using the TCGA-LGG dataset by Pearson's correlation analysis. **(C)** Indicated cells were stained with PE-labeled anti-AC133 and FITC-labeled anti-CD44 antibodies followed by flow cytometry analysis (n=3). **(D)** The relative expressions of miR-6760-5p and SRSF1 in spheroid- and adherently- cultured U251 and A172 cells, respectively, were examined by RT-PCR. **(E)** The correlations between miRNA-6760-5p and OCT4, and CD44, were examined using the TCGA-LGG dataset by Pearson's correlation analysis. **(F)** The correlations between SRSF1 and CD44, NANOG, OCT4, and C-MYC were examined using the TCGA-LGG dataset by Pearson's correlation analysis. **(G-H)** The tumorsphere numbers were quantified after overexpressing miR-6760-5p mimics or SRSF1 targeting shRNAs in GSC11 and GBM1 cells, respectively. NC and the control shRNA were used as reciprocal control, respectively. NGSC=non glioma stem-like cells=parental adherent cells; GSC=glioma stem-like cells= spheroid cultured cells. * $P < 0.05$, ** $P < 0.01$, *** $P < 0.001$.

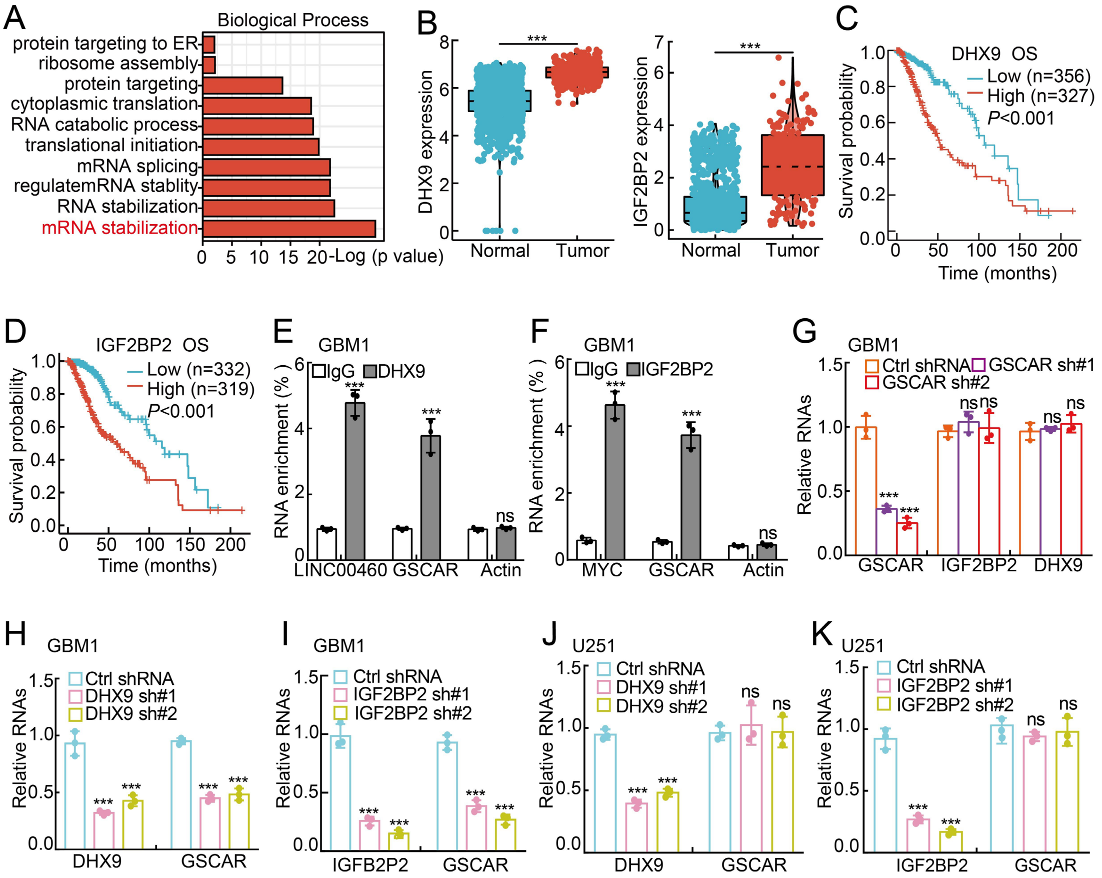

Figure S6 GSCAR mediated the interaction between DHX9 and IGF2BP2. (A) The biological process involved in GSCAR interaction proteins was indicated examined with GO analysis. **(B)** DHX9 and IGF2BP2 were highly expressed in gliomas examined by the TCGA and GTEx datasets (Normal: 1152 and Tumor: 523). **(C-D)** The OS of glioma patients with different DHX9 (C) or IGF2BP2 (D) expressions in the TCGA datasets. **(E-F)** The protein-RNA interaction was verified by RIP assay in GBM1 cells. LINC00460, β-actin, or c-MYC were used as correlative control, respectively. **(G)** The relative expressions of GSCAR，IGF2BP2，and DHX9 after GSCAR knockdown in GBM1 were examined by RT-PCR. **(H-K)** The relative RNA expressions of indicated genes were examined by RT-PCR. * $P < 0.05$, ** $P < 0.01$, *** $P < 0.001$.

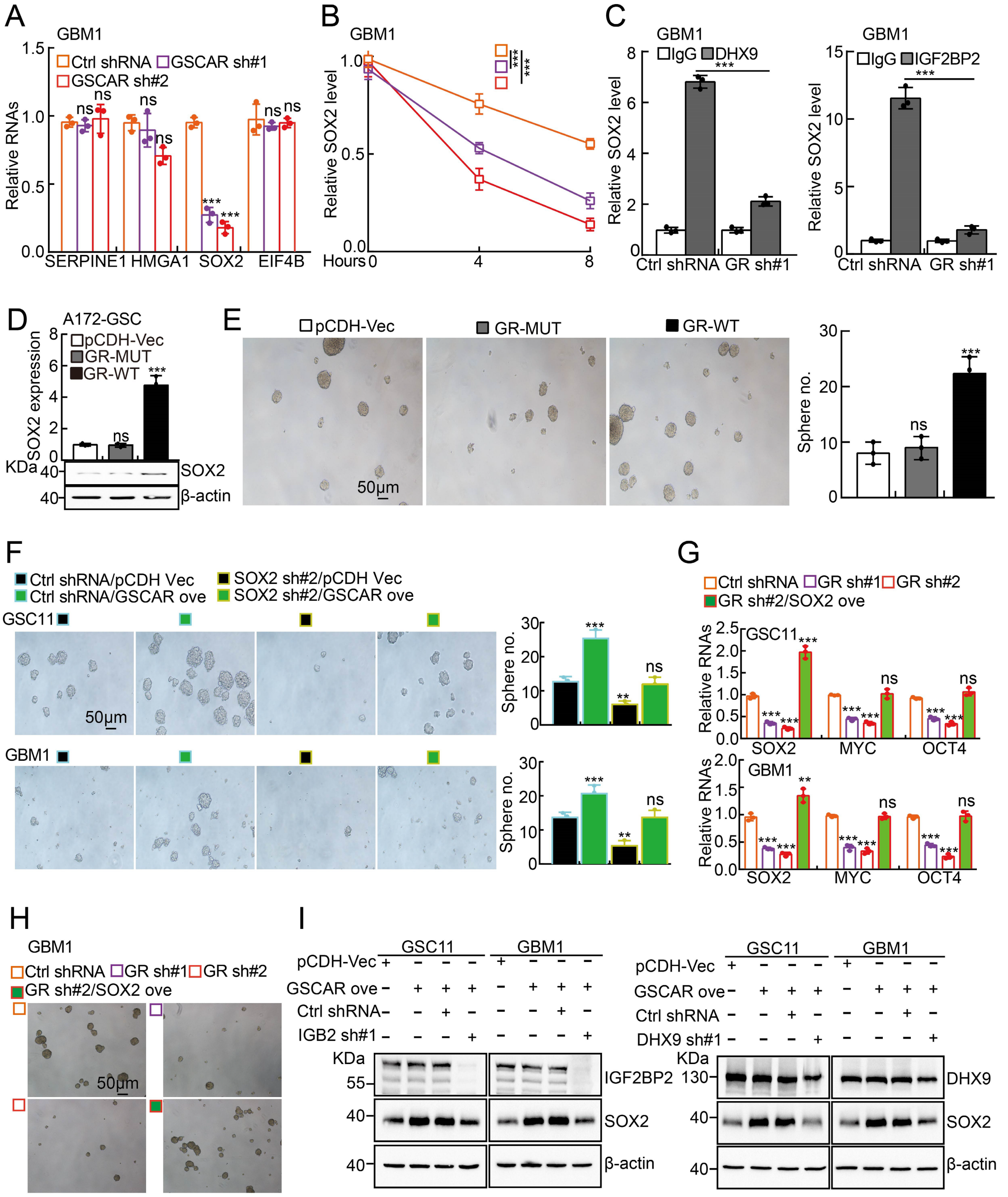

Figure S7 GSCAR promoted DHX9/IGF2BP2 complex formation leading to the stabilization of SOX2 mRNA. (A) The relative expressions of indicated genes after GSCAR knockdown were examined by RT-PCR in GBM1 cells. **(B)** The decay rate of SOX2 mRNA after actinomycin D (5 µg/ml) treatment in GSCAR knockdown GBM1 cells, the scramble shRNA was used as control. **(C)** The protein-RNA

interaction was verified by RIP assay in GBM1 cells after GSCAR knockdown, compared to the control shRNA group. **(D-E)** Forced expression of GSCAR wild-type but not GSCAR mutant missing nt 226 to 475 fragment in A172 cells increased SOX2 expressions examined by RT-PCR and immunoblot (D), and tumorsphere formation ability (E). Quantification results were also indicated. Scale bar: 50μm. **(F)** GSCAR forced-expression reversed SOX2 knockdown effect using tumor sphere formation assay in GSC11 and GBM1 cells. Scale bar: 50μm. **(G)** SOX2 overexpression promoted Oct4 and c-Myc expressions upon GSCAR knockdown examined by RT-PCR. **(H)** Representative images for the tumorsphere formation assay in GBM1 cells. **(I)** The immunoblot assay was performed to detect indicated protein expressions in indicated cells. GR sh#1=GSCAR shRNA#1, * $P < 0.05$, ** $P < 0.01$, *** $P < 0.001$. pCDH-Vec=pCDH lentiviral plasmid vector control. GR-MUT=pCDH-GSCAR mutant, GR-WT=pCDH-GSCAR wild-type. IGB2=IGF2BP2.

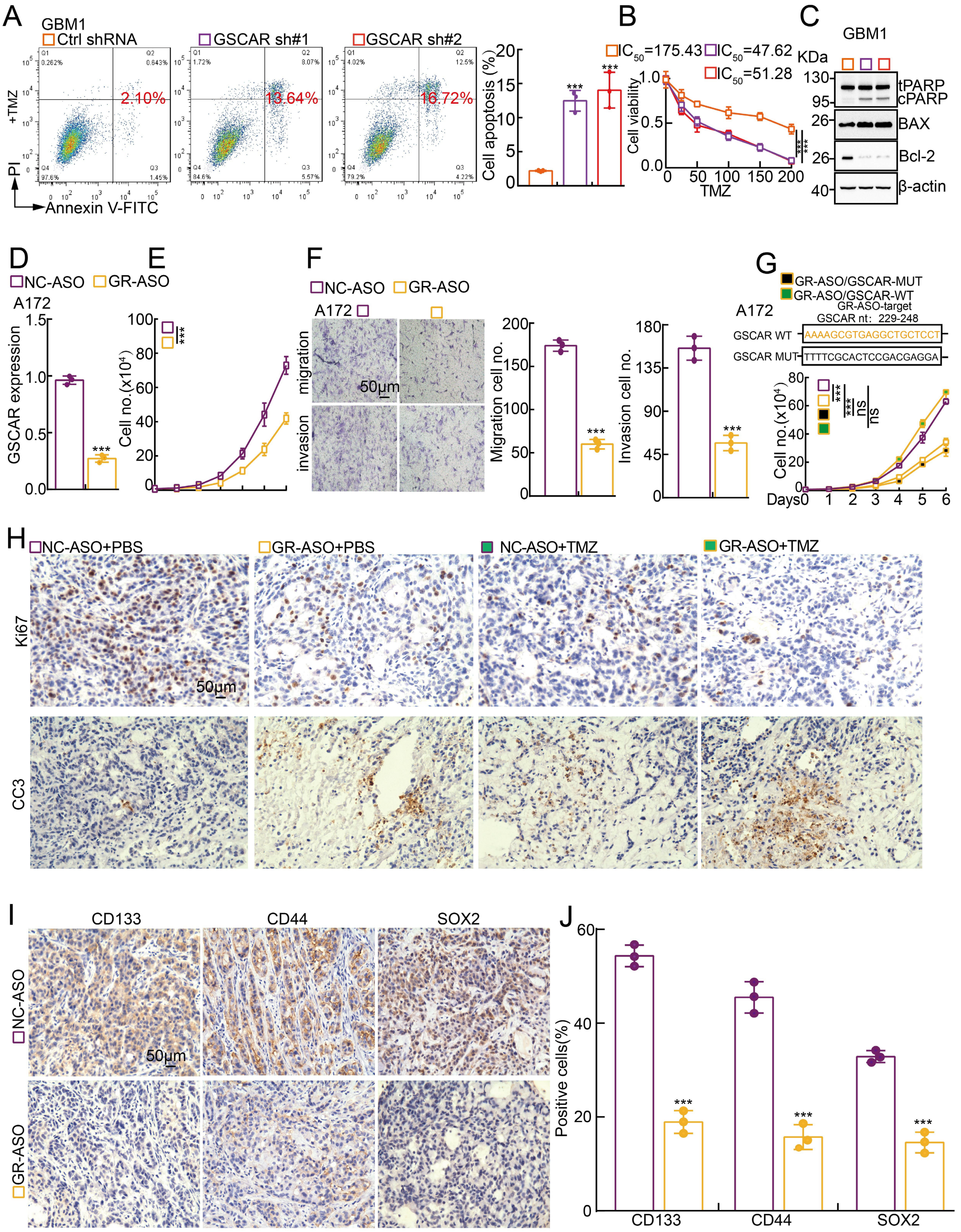

Figure S8 GSCAR targeting ASO reduced tumor growth. (A-B) GSCAR knockdown promoted TMZ-induced cellular apoptosis in GBM1 cells detected by flow cytometry (A) and SRB (B) assays. Quantification results were indicated. **(C)** Marker genes related to cellular apoptosis were detected by immunoblot with

indicated antibodies. **(D)** The relative expression of GSCAR was examined by RT-PCR after indicated ASO transfection. **(E-F)** GSCAR targeting ASO inhibited cell proliferation, migration, and invasion examined by growth curve **(E)** and transwell assay **(F)**. Quantification results were indicated. Scale bar=50 μm. **(G)** GSCAR wild-type but not mutant was able to rescue GSCAR-targeting ASO reduced cell growth phenotype. GSCAR-WT=GSCAR wild-type; GSCAR-MUT=GSCAR mutant=GSCAR targeting ASO insensitive mutant.**(H)** Representative IHC staining images of Ki67 and CC3 for the xenograft tumor sections in figure 8N. Scale bar=50 μm. **(I-J)** Representative IHC staining images of CD133, CD44, and SOX2 for indicated xenograft tumors. (J) Quantification data for (I). Scale bar=50 μm. * $P < 0.05$, ** $P < 0.01$, *** $P < 0.001$.

Supplementary Tables. S1 to S7

Table S1. Antibodies and oligos used in this study.

Antibody Name	Catalog number	Dilution	Supplier	Species
CDK2	10122-1-AP	1:2000	Proteintech	Rabbit
CDK6	ab124821	1:2000	abcam	Rabbit
Cyclin D1	60186-1-1g	1:1000	Proteintech	Mouse
β-actin	60008-1-1g	1:5000	Proteintech	Mouse
p27	610241	1:2000	BD	Mouse
E-cadherin	ab40772	1:500	abcam	Rabbit
N-cadherin	ab18203	1:1000	abcam	Rabbit
Vimentin	103661-1-AP	1:2000	Proteintech	Rabbit
PARP	9542S	1:1000	CST	Rabbit
Cleaved caspase3	9661S	1:500	CST	Rabbit
Bcl-2	15071S	1:500	CST	Mouse
Bax	ab77566	1:1000	abcam	Mouse
β-catenin	610153	1:2000	BD	Mouse
SRSF1	12929-2-AP	1:1000	Proteintech	Rabbit
Oct4	ab19857	1:1000	abcam	Rabbit

Sox2	11064-1-AP	1:1000	Proteintech	Rabbit
DHX9	17721-1-AP	1:1000	Proteintech	Rabbit
IGF2BP2	11601-1-AP	1:1000	Proteintech	Rabbit
CD133	ab19898	1:1000	abcam	Rabbit
CD44	3570s	1:1000	CST	Mouse
**Oligo name**	**Primer sequences (5'-3')**			
β-actin_F	AAGTGTGACGTGGACATCCGC			
β-actin_R	CCGGACTCGTCATACTCCTGCT			
Sox2_F	CACAGATGCAACCGATGCA			
Sox2_R	GGTGCCCTGCTGCGAGTA			
Oct4_F	CTGGGTTGATCCTCGGACCT			
Oct4_R	CCATCGGAGTTGCTCTCCA			
GSCAR_F	GGGTCACAGGGCCACACCG			
GSCAR_R	CAAGGGCATACTTGAGCCTA			
c-Myc_F	GGCTCCTGGCAAAAGGTCA			
c-Myc_R	CTGCGTAGTTGTGCTGATGT			
CyclinD1_F	GCTCCTGTGCTGCGAAGT			
CyclinD1_R	TGTTCCTCTCAGACCTCCAG			
SRSF1_F	GCCGTATTTGTAGAACACGTCCT			
SRSF1_R	CGAACCATCTCAGCGACAAAA			

DHX9_F	TGAGGTCCATGCTTATTTGCTC
DHX9_R	GACAATGGCGATGACCACTCA
GSCAR_F	CACACCGAAAGCCCTCATCT
GSCAR_R	GTAAGAGCGAAACGTTGCCC
IGFBP2_F	CAGCTCCTTCATACCCGACTT
IGFBP2_R	CCTGAATCTCTGGTACGACTGC
EMP3_F	GCCATTCTCGCTGACATTACTG
EMP3_R	CCTGGGCACTTACAGGAAGG
SERPINE1_F	GGTCCGATTCGTCGTCAAATAAC
SERPINE1_R	AGCGAAGTGCCAACACCTAAG
HMGA1_F	TGGTGGTTTTCCGGGTCTTG
HMGA1_R	AGGGGAAGACTATCTCCCTAACA
EIF4B_F	TCATCCGTTTCATCAGCCCAG
EIF4B _R	GCCGTATTTGTAGAACACGTCCT
LINC00460_F	ACGCAGTGGATGAGAACGAA
LINC00460_R	GGGGTGACTTCAGAATGCGT
18S rRNA	GTAACCCGTTGAACCCCATT

-F	
18S rRNA -R	CCATCCAATCGGTAGTAGCG
pGL3-GSC AR-F	CTGAACAGGAACCATTAGAGAA
pGL3-GSC AR-R	AGAGGTCGGGAAGCTGCTGTAA
SBS1-F	TCAATGCCTAGAGCTTA
SBS1-R	AAGGTGGAAAATTGTT
SBS2-F	ATGCTCACAGGACGGTT
SBS2-R	TACAGAGCGAGACTCCG
SRSF1 shRNA	shRNA#1: GCAACCACGAAACCTGTAATA<br>shRNA#2: ACTTACCTCCAGACATCCGAA
IGF2BP2 shRNA	shRNA#1: AGTGAAGCTGGAAGCGCATAT<br>shRNA#2: GGTGCCTGCAGCGGTAATATA
SOX2 shRNA	shRNA#1: CAGCTCGCAGACCTACATGAA<br>shRNA#2: CTGCCGAGAATCCATGTATAT
DHX9 shRNA	shRNA#1: GAAGGATTACTACTCAAGAAA<br>shRNA#2: CCAGAAGAATCAGTGCGGTTT
Hsa-miR-67	CAGGGAGAAGGUGGAAGUGCAGA

60-5p_qPCR	
Hsa-miR-67 60-5p mimics	CAGGGAGAAGGUGGAAGUGCAGA<br>UGCACUUCCACCUUCUCCCUGUU
Hsa-miR-67 60-5p inhibiitor	UCUGCACUUCCACCUUCUCCCUG
U1_qPCR	CCATTGTACTCAGTATGTGCTGACTG
GSCAR ASO	AAAAGCGTGAGGCTGCTCCT

Table S2. The pathological characteristics of patients with gliomas and health donors.

Patients characteristics	No. (%)
**Glioma tissues**	60
**Age(years)**	
≤50	20 (33.3%)
＞50	40 (66.7%)
**Gender**	
Male	21 (35%)
Female	39 (65%)
**Normal brain tissues**	10
**Age(years)**	
≤50	4 (40%)
＞50	6 (60%)
**Gender**	
Male	5 (50%)
Female	5 (50 %)

Table S3: The candidate proteins interacting with GSCAR detected by RNA pull down assay and Mass spectrometry analysis.

Gene names	Protein name	Unique sequence coverage [%]	Mol. weight [kDa]	Score	Intensity GSCAR antisense	Intensity GSCAR sense
**IGF2BP2**	**Insulin-like growth factor 2 mRNA-binding protein 2**	**192.1**	**66.785**	**12.551**	**0**	**124768700**
SNRPD2	Small nuclear ribonucleoprotein Sm D2	12.8	8.7769	2.2032	0	3841800
RPS21	40S ribosomal protein S21	12.3	8.85	2.5636	0	213000
RPL27A	60S ribosomal protein L27a	12.1	10.127	1.8095	0	789000
RPL35A	60S ribosomal protein L35a	12.8	10.645	2.4671	0	374900
RPL36	60S ribosomal protein L36	17	10.789	1.6596	0	19100
**DHX9**	**ATP-dependent RNA helicase A**	**123.43**	**140.96**	**18.24543**	**0**	**138140300**
RAVER1	Ribonucleoprotein PTB-binding 1	13.5	10.831	1.8876	0	539400
EIF3H	Eukaryotic translation initiation factor 3 subunit H	41.3	4.3186	1.5402	0	1185700
RPS14	40S ribosomal protein S14	16	16.159	4.1487	0	6721000
DDX6	Probable ATP-dependent RNA helicase DDX6	5.9	20.426	1.8095	0	439800
GNB2L1	Guanine nucleotide-binding protein subunit beta-2-like 1	20.7	21.934	5.8262	0	372000
SNRPB2	U2 small nuclear ribonucleoprotein B	4.9	25.486	1.5527	0	38800

VAPA	Vesicle-associated membrane protein-associated protein A	4.8	27.893	1.5223	0	576000
EIF3I	Eukaryotic translation initiation factor 3 subunit I	3.7	36.501	2.425	0	46100
LIMA1	LIM domain and actin-binding protein 1	5.9	43.19	4.3146	0	166000
EIF3E	Eukaryotic translation initiation factor 3 subunit E	4.5	52.22	2.2913	0	933000
G3BP2	Ras GTPase-activating protein-binding protein 2	8.7	54.12	10.111	0	754000
PUF60	Poly(U)-binding-splicing factor PUF60	4.8	54.626	1.899	0	43900
EIF3L	Eukaryotic translation initiation factor 3 subunit L	3.4	55.161	5.5782	0	133000
HNRNPR	Heterogeneous nuclear ribonucleoprotein R	2.6	55.717	2.326	0	53300
TRMT5	tRNA (guanine(37)-N1)-methyltransferase	2	58.246	1.362	0	4000
YTHDF3	YTH domain-containing family protein 3	1.7	58.311	1.749	0	44800
SBSN	Suprabasin	6.1	60.54	4.2106	0	128000
RAIL4	Neurofilament light polypeptide	5.3	110.04	4.3786	0	3000
CTTN	Src substrate cortactin	6	61.585	6.0245	0	134000
ABCF1	ATP-binding cassette	2.5	67.55	2.422	0	62900

	sub-family F member 1					
PPP1R12A	Protein phosphatase 1 regulatory subunit 12A	3.9	76.532	5.0972	0	46700
EIF4G1	Eukaryotic translation initiation factor 4 gamma 1	2.8	82.494	2.637	0	158000
PKP1	Plakophilin-1	2.9	82.86	5.2573	0	190000
EIF3B	Eukaryotic translation initiation factor 3 subunit B	1.6	92.48	2.6706	0	79200
EIF4G2	Eukaryotic translation initiation factor 4 gamma 2	3.6	98.117	5.9513	0	10000
SUCO	SUN domain-containing ossification factor	0.9	98.283	2.353	0	40600
EIF3C;EIF3CL	Eukaryotic translation initiation factor 3 subunit C	2.6	105.34	2.3383	0	198000
RAI14	Ankycorbin	4	110.04	10.099	0	91900
CCDC146	Coiled-coil domain-containing protein 146	1	112.81	2.1906	0	88000
INTS3	Integrator complex subunit 3	1.1	118.07	1.7656	0	28000
MYO1C	Unconventional myosin-Ic	3	118.99	3.3546	0	8600
COL1A1	Collagen alpha-1(I) chain	0.6	138.94	1.708	0	0100
EIF3A	Eukaryotic translation initiation factor 3 subunit A	5.2	166.57	16.412	0	469000
PRRC2C	Protein PRRC2C	0.5	308.77	5.632	0	2600

Table S4: The candidate lncRNAs were identified by integrative omics analyses using various TCGA-LGG datasets.

TCGA-LGG	GSE146698	GSE131744	GSE188256	
FOXCUT	AC092718.4	LINC01914	MIR4435-2HG	AP001107.6
PHEX-AS1	MIR3681HG	STRA6LP	AC023043.4	AC025176.1
AC007098.1	LINC02709	AC022007.1	LINC01508	PRKR1B-AS1
AC027237.3	AP001107.6	PVT1	AL451042.2	AC007344.1
PRECSIT	PVT1	AC099850.3	MIR155HG	AC010524.1
AL512353.1	AL512353.1	AC103746.1	AL512353.1	AC009950.1
AC010271.1	AC010271.1	CRNDE	AL512353.1	ZNF236-DT
LINC01579	LINC01579	**AC008525.1**	AC010271.1	LINC00601
PVT1	LINC01060	AC010255.1	**AC008525.1**	LINC01060
AP000697.1	AC009495.3	C1RL-AS1	LINC02777	MIR1915HG
AL606970.4	CRNDE	AC004943.2	LINC01995	AL604028.1
AC005790.1	LINC01010	LINC01060	AP001025.1	THORLNC
NRIR	AL050403.2	LINC02709	LINC01127	PVT1
AL117332.1	LINC01206	AP001107.6	LINC00706	PAXIP1-AS2
TGFB2-AS1	AC090579.1	AL139383.1	CTD-221I18.1	LINC01914
LINC01060	LINC01956	PTCHD1-AS	AC093523.1	SU4-STRA6LP
AL604028.1	AP001610.2	FKBP14-AS1	RHPN1-AS1	AC022007.1
THORLNC	AC010255.2	FLJ12825	AC127502.2	AC106786.1

AC104574.2	**AC008525.1**	AC009961.1	CRNDE	AL512329.2
EPBL4A-DT	MIR155HG	AC002398.2	AC108047.1	AC108463.2
AC093673.1	AC087501.4	AC023983.1	AC008972.2	AC010168.2
**AC008525.1**		AP002761.4	DTX2P1-UPK	AC068631.1
GNAS-AS1			AC009336.1	ELDR
CRNDE			AC108463.3	AC139149.1
			LINC01579	AC087477.2
**AC008525.1-References**	**No**			
LINC01060-References	DOI:10.1158/0008-5472.CAN-20-2270,	DOI: 10.1016/j.canlet.2018.06.015,	DOI: 10.1097/FPC.0000000000000302	
PVT1-References	DOI: 10.1007/s13311-018-0649-9,	DOI: 10.26355/eurrev_202008_22590	DOI: 10.26355/eurrev_202008_22590	
CRNDE-References	DOI: 10.1155/2021/7566365	DOI: 10.1016/j.canlet.2015.03.027	DOI: 10.1186/s12935-021-02153-x	

AC008525.1 (ENSG00000250377): GSCAR

TCGA-LGG: Differential gene expression analysis in LGG, somatic copy number alterations (SCNAs) in LGG, and 24 candidate lincRNAs were selected according to the criteria (relative CNAs in >40% glioma samples, occurring in the amplification CNA area, prior to long intergenic non-coding RNA, Log FC>4, p<0.0001).

GSE146698: Differential gene expression analysis between adherent U87MG cells and U87MG cell-derived glioma stem-like cells (Log FC>4, *P*<0.0001).

GSE131744: The gene expressional profiles between U87MG glioma cells and the derived TMZ-resistance cells (Log FC>4, *P*<0.0001).

GSE188256: Expression data from human glioma tissues and corresponding adjacent non-tumor tissues (Log FC>4, *P*<0.0001).

Table S5: The predicted GSCAR downstream targeted miRNAs examined by StarBase, LncBase V2 and Annolnc2, respectively.

StarBase	LncBase V2	Annolnc2
MIR8081	MIR515-1	MIR6128
MIR-6760-5p	MIR607	MIR3179-3
MIR378D1	MIR4672	MIR4253
MIR6131	MIR3156-1	MIR4427
MIR4740	MIR378C	MIR4309
MIR129-2	MIR6134	MIR190B
MIR6720	MIR2115	MIR5701-2
MIR516A2	MIR520G	MIR1302-8
MIR548C	MIR-942-5P	MIR433
MIR638	MIR378A	MIR-942-5P
MIR548AM	MIR548U	MIR6833
MIR574	MIR6129	MIR6089
MIR569	MIR3665	MIR4791
MIR2681-5p	MIR596	MIR6129
MIR217	MIRLET7F2	MIR548AP
MIR6737	MIR6777	MIR6071
MIR4421	MIR2113	MIR4290
MIR519D	MIR7976	MIR3176

MIR1323	MIR6870	**MIR-6760-5p**
MIR5194	MIR190A	MIR507
MIR4518	MIR6859-2	MIR4686
MIR6865	MIR3943	MIR4665
MIR4659A	MIR135A2	MIR2681
MIR8075	MIR1299	MIR3923
MIR7850	MIR1260A	MIR4435-2
MIR5000	**MIR-6760-5p**	MIR6864
MIR6815	MIR421	MIR5580
MIR194-1	MIR2681-5p	MIR6806
MIR4266	MIRLET7E	MIR1273C
MIR4635	MIR6806	MIR7853
MIR6129	MIR4659A	MIR6809
MIR3117		MIR3165
MIR-942-5P		MIR3118-2
MIR3176		MIR4676
		MIR660
		MIR4654
		MIR3684
		MIR2681-5p
		MIR1238

Table S6: The predicted downstream target genes of miRNA-6760-5p examined by StarBase, miRDB, miRGator and miRWalk, respectively.

StarBase	miRDB	miRGator	miRWalk	
C6orf223	TLDC2	OR2T34	TMOD2	TRMT5
AL133373.1	CLEC1B	EYA3	ROGDI	EIF4EBP2
PRRT2	PTMS	CBLN1	FOSB	SHISA6
POU2AF1	ZNF703	PEF1	SEC31B	PPP2R4
CYB561D1	OR5AN1	PCYT1A	RAB1B	GPATCH2L
URM1	CALN1	PDSS2	CD3E	LINC00632
KRTAP4-8	PPP1R12B	TIPARP	DCTN3	AC110619.2
WDTC1	KLRF1	CECR1	C3orf72	DNAJC5G
VAMP2	SCN4B	CHST14	BCL2L13	GOLPH3L
NXF1	MLLT11	DLX6	USP2	MICAL2
TMEM9	VAPB	MVB12B	TADA3	BTD
CCL22	ZFAND2B	CASP14	BTG2	SLC9A8
GOSR1	PGBD2	SYT2	PPP3R2	IGFBP2
ZNF444	KRTAP4-11	ICOS	TMEM164	NHLH1
TP53I11	CNOT4	SMAP2	DUT	GMPPA
KIAA1045	HOXC6	AJAP1	CFL1	PACS1
MT-ND4L	MAP4	LRTM2	SPRY4	PHF5A
C20orf96	LASP1	DLX3	DNAJC8	SEMA4G

TPP1	ANKRD45	CYP2C18	SEPT2	ZNF385B
LY6G6C	SCAMP5	PTER	SBK1	RPGR
SLC25A44	PIP4K2C	SH2D1B	ZFP36L1	TFRC
ZNF385A	C17orf78	ZNF660	COG1	DMRTA1
NOVA2	CHCHD5	DCLK1	SIX5	GCSAM
PAX1	XCR1	DUSP7	CD84	POU2F2
DMBX1	IDH3G	APLNR	DTX1	FLJ00104
PDX1	LHFPL3	GJA5	JMJD7-PLA2G4B	C9orf78
CSAG1	BAZ2A	TP53INP2	CHORDC1	**SRSF1**
SCN2B	PPM1M	ALPK3	KCNE3	MED23
SIX2	RP11-863K10.7	PDK2	EIF1	HOXA10
DYNLL2	DACT1	FAM203A	MADCAM1	GDA
CTD-2616J11.4	ADAM19	SLAMF7	OPTC	CCDC97
IGFBP2	GABBR2	DDAH1	AKTIP	CECR6
ADO	ACTR3C	DUSP13	ZBED3	EMP3
SNX12	**SRSF1**	AL020996.1	COPZ1	C17orf77
SPATA12	PIANP	KIAA1671	RP11-1102P16.1	CDK5R2
**SRSF1**	ANAPC5	PATE3	PLEKHH2	HS3ST2

AC117834.1	EMP3	CRY2	DLX4	SHISA7
PPARD	RSPO4	MINOS1-NBL<br>1	AL645730.2	GABRE
PABPC1L2A	POLE3	OSBPL7	CD300E	TRIM4
CERS2	AP000769.1	CCDC113	KRTAP9-7	PBX2
RPS2	KLK4	UBE2D2	AP001579.1	CYP19A1
RSPO4	ERP27	VCPIP1	SURF4	GPR111
POLE3	S100A16	AC007040.11	FOSL1	RASSF3
DDAH1	CALM2	NPNT	CACNG6	IGF2BP1
DUSP13		PCDHB13	ST8SIA3	FLJ45079
AL020996.1	KRTAP9-9	PROSC	CDPF1	COLEC10
KIAA1671	CACNG4	GAP43	HINT2	C21orf128
UBQLN4	TTC34	PRR15L	ATP6V0D1	SMARCD1
EMP3	ARL4A	MPZL1	ZNF571	CACNG7
TRAM2	IGFBP2	IGLL5	IKBKE	DEFB134
	CASP2	PEA15	CDH23	MXD1
	B4GALT4	GJB4	CLEC4G	DUOX1
	WNT8B	TMEM86A	TMEM97	CD34
	COL28A1	RNF185	NRSN2	ANKRD52
	HTR4	TDO2	IKZF4	RAB3B
	LIMS2	EMP3	CREB3L2	C10orf11

	AP001579.1	COL28A1	ZNF391	SNRNP25
	SURF4	HTR4	DCX	LAMTOR4
	FOSL1	LIMS2	PSMD9	RNF165
	ZNF24	RIC8B	PARVA	RPTN
	STK40	SLC6A17	PRELP	CEP128
	RPS2	SHF	TMEM229B	IL2RG
		APOL6	SEPT3	TMEM217
		IL17RD	SLFNL1	SLC17A7
		HOXB5	C12orf43	SSX3
		STAG3	GNAL	DYNAP
		ZNF618	SNX33	ICAM3
		HIP1	PHF12	SCN3B
		ZIC2	ZNF215	MAX
		CNOT7	ADIPOR2	RALGPS1
		NBL1	PIGS	SLFN12
		PIN1	LBH	PYCRL
		GATAD2B	ARL6IP1	OR56B4
		IGFBP2	NPLOC4	NEK5
		PRX	TBX19	LUZPP1
		DIRC1	SEC14L3	KRT6C
		KPRP	FLOT2	TMPRSS5

		ARMC10	AC074091.13	LINGO1
		GRN	B3GAT1	MAP1LC3B
		INSL5	CXorf36	LTBR
		**SRSF1**	NAALADL1	HOXC12
		NEUROD1	CDK14	C9orf163
		C11orf34	PSMG4	CPLX4
		IKBKG	CYHR1	TPM2
		RPS2	C15orf41	OR2T33
		RSPO4	CLCN6	MON1B
		POLE3	LL22NC03-63E9<br>.3	FAM203B
			C1orf87	HMGA2

Table S7: The candidate targeted genes for GSCAR-DHX9/IGF2BP2 axis identified by multiple web-source available datasets.

GSE192792	GSE31095	GSE83822	TCGA/LGG
BCAN	PRTG	HNRNPU	ACOX1
ASCL1	SALL2	WDR6	ZNF76
RFTN2	FUBP3	TAF4	AP3M1
**SOX2**	ZNF572	ZNF319	LBXCOR1
LOC645323	YTHDC1	ZNF711	PHF12
VANGL2	DARS	ZFAND3	ZNF830
SOX21	**SOX2**	VPS52	PDCL
FAM181B	NKAIN4	UHRF1	PCF11
OLIG2	PHLPP1	ZCCHC18	ZNF606
JAKMIP2	MBTD1	CYTSB	MKRN1
SOX6	CBS	**SOX2**	ASB3
POU3F3	PHF21A	MGC21881	EIF4B
NOVA1	CECR2	EIF4B	SBK1
CRB1	ZFP62	ZNF275	**SOX2**
NPAS3	TMCC1	PCDHB10	ACAD8
C1orf61	C2orf68	WNT3	ZNF687
EIF4B	EIF4B	HMGA1	ZNF608
HMGA1	C1orf94	PLXNB1	LOC643763

DCLK2	DSCAM	SPIRE1	LHFPL4
PCDHB9	LAMB2L	MBIP	HNRNPL
ENAH	NCBP2	SAMD1	CIAO1
OLIG1	HMGA1	C17orf100	PLK1S1
MSI1	NRG2	TSPAN3	MTA2
NFIX	FAM120C	GRHL3	HMGA1
SERPINE1	REPIN1	FMN2	SETDB1
LRRC37B2	DPF2	SERPINE1	SETD2
MKRN3	LOC154822	RND2	BBS2
POU3F2	SERPINE1	GPT2	SERPINE1
TCF7L1	UST	LUZP2	RANBP3
MARCKS	PDE9A	STK17B	EDNRB
CHST9	C10orf2	KDM4B	LOC646999
TCF12	BRD7	SAFB2	ZNF254
QKI	PHC1	SULT1C4	ANKRD13B
EIF3B	VEZF1	HFM1	HNRPDL
MYEF2	FYN	ARHGEF6	SPON1
CHD7	ARHGEF10L	UNK	GNAO1
KCTD5	HEPACAM	THAP9	RNF39
DLL1	EIF3B	NTRK3	METT10D
GAB1	CPSF7	TOP2B	TEAD1

SHD	C6orf134	UNC5CL	ZNF775
NCAM1	MCART6	PGBD1	DST
NTN1	PURG	GPR56	C2orf27A
PTPRZ1	GRIK4	CTTNBP2	MXI1
BCHE	ZSCAN21	IMPDH2	DDX31
PATZ1	ADAMTS6	MAP1D	PATZ1
SOX4	CLNS1A	CSNK1E	SOX4
NOTCH1	NRARP	HNRNPUL1	NOTCH1
NFIB	LOC220930	E2F5	P2RX7
KHSRP	RAF1	HIRIP3	CDC5L
ZBTB12	OPHN1	BAZ1A	INTS4
DPYSL3	APOM	FAM76B	OFD1
MANEAL	SKA2	ZNF624	SMOC1
DCAKD	ILF3	TUBB2B	SNHG1
C14orf93	CRMP1	HNRNPA1	DDX19B
RAF1	TFAP4	NKX2-2	GOLIM4
OPHN1	H3F3B	CACNG7	NLGN2
APOM	ZNF610	RAF1	BAT2
BEND7	ANKFY1	OPHN1	HNRNPA0
PMP2	JAM2	APOM	DLG5
TCF3	PRR14	ST7	NOL8

ANKFN1	WHSC2	BRD3	AKAP1
GNRHR2	CDH20	KCNN2	CDKN1B
MAGEF1	FAM84B	NONO	SYTL4
RFX4	ZNF19	CCDC88A	GNA13
PHF21B	KDM3B	KIAA1267	BCL6
VCAN	TROVE2	MYCL1	DPYSL4
ZNF853	SOX2OT	CHD4	SALL3
DLL3	SETD5	ANTXR1	FUT3
RHOBTB3	SOX3	FAM192A	C19orf54
NEU4	ZNF501	RHOBTB3	SAP30BP
MEX3A	NKD1	NEU4	CEP57
LOC729991-MEF2B	SALL1	CTCF	REXO4
ARL6IP6	DCAF12	ARHGAP31	ZNF389
GPSM2	SF3B2	USP10	LOC100190939
SRGAP1	DENND5A	UBQLN4	ADCY8
AATF	SEMA6A	PTCHD2	HMGN5
ZNF193	SEMA5B	BTBD17	KEAP1
MKS1	GLCCI1	CASK	FAM35B2
NLGN1	HNRNPH3	AATF	CWC27
MASP1	GKAP1	ZNF193	KIAA1549
GLDC	MARCKSL1	MKS1	ZBTB20

PHF16	ZBTB5	ZNF227	HTATSF1
LOC283761	JRKL	SIAH1	FUS
ST7OT1	MAPT	PHF17	ATAD2B
APC2	IFT140	GATS	AXIN1
LOC254559	ZBED1	TLE3	LRP5
LRIG1	FXYD6	NOL4	CHERP
TRIM9	FERMT1	ZNF253	SYT6
	KCTD15	HNRNPK	BAZ2B
	LRRN1	NTRK2	SMAD5
	NAB1	SEPHS1	RBM12B
	MSTN	TRIM39	MEGF11
	ETV1	HEATR6	FUT5
	TFCP2	ZNF24	TARDBP
	EPB41L5	GPC2	CDH10
	TMEM170A	STAG2	ANGPTL2
	MFF	TCF4	VPS37D
	MAML2	TSEN2	NMB
	GBX2	ZNF74	SGEF
	ZNF197	LOC144486	SGPL1
	C2orf88	RAVER1	ZSCAN16
	AGPAT5	HEY2	ZNF493

	PAG1	ZNF621	RBBP6
	PRKX	LRRC1	LOC84989
	DPP6	ZXDC	CRYAB
	ZNF187	SFT2D3	LUC7L3
	PKN1	FAM35B	HNRNPR
	FLJ16779	HES5	C1QL1
	PHF2	GSTA4	PABPC1
	CWF19L2	PTPRS	YEATS2
	HNRNPA1L2	MTSS1L	CCDC66
	FCHSD2	KIF7	MYST2
	ZNF100	MIDN	MYC
	SPAG9	STAMBP	RELA
	TRMT5	FRMD5	CALCRL
	EEF1G	DHTKD1	GALNT13
	GTF2IRD2P1	ZNF250	RGS9
	SCG3	SMARCC2	ABL1
	TAOK3	NUMA1	ZNF3
	GRIA4	FAM193A	ZNF664
	CBX1	TLK2	ZNF559
	C10orf25	CUEDC1	NFRKB
	ZEB1	SOX11	CCDC41

	C11orf57	CSMD2	C2CD3
	TIGD1	RFX7	C5orf54
	NCKAP5	OR4N2	APBB2
	CTNND2	SPATA5	ZNF337
	MTMR1	LIX1	MED17
	DVL2	TOX3	POLDIP3
	EBF4	GRIA3	PRRX1
	SCN3A	CACNG4	UBN2
	CASC3	MAP2	NLGN3
	C7orf61	CADM4	RBM10
	MSL2	C17orf69	LOC400027
	ZNF696	MTA1	ZNF285
	ZNF738	CORO1C	ZNF491
	RIPK2	KLHL25	PBX1
	LARS	RABGAP1	POLA1
	SNRNP200	SMAD1	PAX6
	CPNE2	ZSWIM5	GADD45G
	OSGEPL1	PRSS27	PALB2
	MED24	NCRNA00171	ADCY2
	DDX6	GOLGA1	PM20D2
	CASP9	FXR1	ZFP37

	STAT5B	LOC284900	LOC147727
	ZNF620	LRRC58	GPR19
	LOC729678	RBM14	MGC57346
	TIGD7	BCL7A	ST8SIA1
	ZNF34	NEK4	C19orf57
	SNCAIP	ZNF512	CBR4
	PTCH1	ZNF212	TTC28
	GTF2F2	HNRNPA2B1	KCND2
	LOC148145	TJP2	TBC1D5
	ZMAT1	TNIK	MTHFSD
	PHF6	ZNF862	SDR39U1
	FTSJ3	TMEM145	RG9MTD3
	ACVR2B	CXXC4	TMEM100
	CTPS2	FAM182B	PEA15
	LPHN3	PPP2R3B	SENP7
	SARM1	PEX12	C12orf34
	PRPF38A	HEY1	
	LOC100128977	PLXNB3	
		HNRNPD	
		LDLRAD3	
		THRA	

		SCD5	
		SMC3	
		EEPD1	
		VIPR2	
		ADD2	
		MAMLD1	
		ADAMTS20	
		SUV420H1	
		TAF15	
		TMPRSS5	
		PSAT1	
		MIF4GD	
		MYST1	
		ZNF490	
		ZDHHC22	
		HNRNPC	
		LOC100128288	
		ZNF317	
		ZC3H4	
		KDM4D	
		SCHIP1	

		LRRC37B	
		ZBTB33	
		PCIF1	
		LIX1L	
		PCDH15	
		PRKD1	
		MED13	
		PRPF40A	
		OSGIN2	
		RNF40	
		NAV2	
		ZBTB10	
		C8orf31	
		RBM4B	
		ZSCAN29	
		POLR1E	
		RAC3	
		PHYHIPL	
		WDR75	
		EPHB1	
		THAP2	

		TSHR	
		C2orf63	
		SBF2	
		QSER1	
		NSUN6	
		KLF15	
		GPBP1	
		C16orf87	
